# Overexpression of TSG101 causes the development of adenosquamous mammary carcinoma

**DOI:** 10.1186/s13058-025-02007-8

**Published:** 2025-07-07

**Authors:** Rayane Dennaoui, Patrick D. Rädler, Madison N. Wicker, Kerry Vistisen, Rosa‑Maria Ferraiuolo, Aleata A. Triplett, Hridaya Shrestha, Tessa A. Liner, Karoline C. Manthey, Hallgeir Rui, Robert D. Cardiff, Teresa M. Gunn, Charles M. Perou, Kay-Uwe Wagner

**Affiliations:** 1 Department of Oncology, Wayne State University School of Medicine and Tumor Biology Program, Barbara Ann Karmanos Cancer Institute, 4100 John R, Mail Code EL01TM, Detroit, MI 48201 USA; 2 Department of Genetics, Lineberger Comprehensive Cancer Center, University of North Carolina at Chapel Hill, Chapel Hill, NC 27599 USA; 3 Eppley Institute for Research in Cancer and Allied Diseases, Nebraska Medical Center, University of Nebraska Medical Center, 985950, Omaha, NE 68198-5950 USA; 4 Department of Biology, Coastal Carolina University, Conway, SC 29528 USA; 5 Department of Pharmacology, Physiology & Cancer Biology, Thomas Jefferson University, Philadelphia, PA 19107 USA; 6 Department of Pathology and Laboratory Medicine, School of Medicine University of California, Davis, CA 95616 USA; 7 McLaughlin Research Institute, and Touro College of Osteopathic Medicine , Touro University, Great Falls, MT 59405 USA; 8 Present Address: Canadian College of Health Science and Technology, 1737 Walker Road, Windsor, ON N8W 3P2 Canada; 9 Present Address: Becton Dickinson, 21 Davis Drive, Durham, NC 27709 USA; 10 Present Address: PrecisionAQ, 777 Township Line Road, Suite 300, Yardley, PA 19067 USA

## Abstract

**Background:**

The mammalian *Tumor Susceptibility Gene 101* (*TSG101*) encodes a protein with diverse functions that control the proliferation and survival of cells, but its role in malignant transformation and cancer development has remained enigmatic.

**Methods:**

To study the pro-tumorigenic functions of TSG101, we developed a bi-transgenic mouse model that expresses exogenous TSG101 along with a luciferase reporter in a ligand-controlled manner in the mammary gland epithelium. We performed a comprehensive histopathologic, biochemical, and molecular characterization of ductal hyperplasia and mammary tumors. Unsupervised hierarchical clustering based on 1,723 intrinsic genes of ten TSG101-overexpressing cancers alongside 251 tissue samples representing 31 reference mammary tumor models and normal mammary glands was conducted.

**Results:**

Females overexpressing TSG101 develop ductal hyperplasia, adenomyoepitheliomas, and palpable adenosquamous carcinomas at an average latency of approximately ten months. These metaplastic mammary tumors are comprised of transforming basal and luminal epithelial cells. Using a GFP reporter strain to monitor the transgene activation at the single-cell level, we determined that the epithelial heterogeneity within transforming ducts and ensuing carcinomas originated from the luminal epithelium. At the molecular level, TSG101-induced mammary tumors are triple-negative and exhibit gene expression signatures of Wnt and inflammatory cytokine signaling, which are key regulators of epithelial cell fate. The ligand-controlled downregulation of exogenous TSG101 in established carcinomas led to tumor regression. We demonstrated that the TSG101-mediated activation of PI3K/AKT signaling, as well as upregulation of Cyclin D1 and MDM2, are dependent on the perpetual expression of the TSG101 oncoprotein.

**Conclusions:**

The collective findings of this study provide in vivo evidence that TSG101 possesses pro-tumorigenic properties that extend to cancer progression and maintenance, suggesting that this protein could be a rational molecular target to prevent and treat a subset of mammary tumors.

**Supplementary Information:**

The online version contains supplementary material available at 10.1186/s13058-025-02007-8.

## Introduction

The *Tumor Susceptibility Gene 101* (*TSG101*) encodes a protein with versatile functions in various molecular and biological processes that are essential for cell growth and survival [1]. *TSG101* is the mammalian ortholog of the yeast class E *Vacuolar Protein Sorting 23* (*VPS23*) [2, 3], and in combination with other vacuolar proteins (i.e., VPS28, VPS37), VPS23/TSG101 forms the core of the Endosomal Sorting Complex Required for Transport I (ESCRT-I). As part of this complex, TSG101 is suggested to selectively bind and control the intracellular movement of ubiquitinated cargos and proteins containing PT/SAP tetrapeptide motifs. Selected cargo proteins are sorted into multivesicular endosomes destined for degradation [4], but TSG101 is also found in exosomes and has been shown to play critical roles in the biogenesis and release of extracellular vesicles [5, 6]. In addition to cellular trafficking, other proposed functions of TSG101 include the regulation of transcription [7, 8], proliferation, and cytokinesis [9–11].

The murine *Tsg101* gene is ubiquitously expressed in all tissues throughout development, and conventional knockout mice die very early during embryogenesis [12–14]. A recent review by Ferraiuolo et al. [1] provides an overview of the biologically significant roles of TSG101 in normal organogenesis and diseases using various genetic models. The Cre recombinase-mediated deletion of both copies of the *Tsg101* gene in primary mouse embryonic fibroblasts causes a cell cycle arrest at the G1 phase that is p53 and p21^Cip1^-dependent [15]. Moreover, TSG101-deficient normal, immortalized, and tumorigenic cells engage p53-independent molecular mechanisms that initiate autophagy and programmed cell death [14–17].

*Tsg101* was initially identified in transformed mouse NIH3T3 cells using an insertional mutagenesis screen [18]. A loss of contact inhibition and colony formation was observed when the *Tsg101* cDNA was expressed in both antisense and sense orientation in NIH3T3 cells, suggesting that this gene might have tumor-suppressive and oncogenic properties. Subsequent studies reported that the TSG101 protein is overexpressed rather than lost in clinical specimens of breast, lung, thyroid, ovarian, and colon cancers [19–24]. Moreover, experiments on human cancer cell lines from patients with breast, prostate, renal, ovarian, and hepatocellular carcinomas demonstrated that TSG101 is required for the growth and survival of malignant cells [23, 25–28]. In genetically engineered mice, the deletion of one or two copies of the *Tsg101* gene does not lead to cancer development on a p53 wild-type or p53-deficient background [13–15]. Similar to human cancer cell lines, the lack of TSG101 prevented the initiation of ERBB2/neu-induced mammary cancer [29]. The notion that TSG101 executes primarily pro-tumorigenic roles in breast carcinogenesis was also supported by immunostaining results that showed that this protein is expressed at higher levels in invasive breast cancer cases [20].

More than two decades after *Tsg101* was first cloned and designated as a tumor susceptibility locus, there are no genetically engineered models that develop cancer in response to the overexpression or downregulation of this gene. The generation of tumor models might have been hindered by the fact that in most cell types, the level of the TSG101 protein is tightly controlled by a posttranslational, autoregulatory feedback mechanism that is still poorly defined [30]. The overexpression of TSG101 outside of this narrow physiological range causes cell death [10]. Cardiomyocytes and differentiated alveolar cells of the mammary gland are the only cell types reported thus far that can tolerate elevated levels of exogenous TSG101 [31, 32]. Transgenic female mice that overexpress TSG101 under the *Whey acidic protein* (*Wap*) promoter in the mammary gland (WAP-TSG101) [32] exhibited a higher incidence of sporadic tumor formation. However, given the low penetrance (18%) and very long tumor latency (1–2 years), the oncogenic potency of TSG101 appeared to be relatively weak. It should be noted that the temporal activation of the WAP-TSG101 transgene in the mammary gland was primarily limited to differentiated alveolar cells during pregnancy and lactation and it remained undetermined whether other epithelial subtypes in the gland are more susceptible to TSG101-induced neoplastic transformation. Here, we report the generation of a bi-transgenic mouse model (MMTV-tTA TetO-Tsg101) that overexpresses TSG101 in a temporally and spatially controlled manner in the luminal epithelium throughout mammary gland development. The collective results of the cellular and molecular analyses of this animal model demonstrate that TSG101 is an oncoprotein, whose upregulation promotes the development of adenosquamous mammary carcinoma with gene expression signatures of high Wnt and inflammatory cytokine signaling. More importantly, the growth and survival of mammary carcinoma cells in tumor-bearing mice were dependent on the sustained upregulation of TSG101, suggesting that this oncoprotein might serve as a rational therapeutic target.

## Materials and methods

### Generation of TetO-Tsg101 transgenic mice

The cloning of a vector expressing the full-length coding sequence of TSG101 with an N-terminal hemagglutinin (HA) tag and its use to functionally rescue a conditional knockout of the *Tsg101* gene was described previously [16]. The *HA-Tsg101* sequence was cloned as a blunted fragment into the *Sma*I site in front of an IRES-Luciferase construct [33], and the *Tsg101-IRES-luciferase* expression cassette was transferred as a blunted *Not*I/*Sac*I fragment into the *Eco*RV site of the pTet-Splice vector. Prior to pronuclear injection into FVB zygotes, the *TetO-Tsg101-IRES-luciferase* transgene was released from the vector backbone using *Xho*I/*Not*I and gel purified. Transgenic founder mice were generated at the McLaughlin Research Institute Transgenic Facility and Cyagen US, Inc. We obtained seven founder animals that were mated with FVB/N wild-type mice to establish individual transgenic lines, and six founders transmitted the TetO-Tsg101 transgene to their progeny. Offspring were screened by genomic PCR with primers recognizing approximately 500 bps of the junction between the IRES and the luciferase coding sequence (forward primer 5′- CCA TTG TAT GGG ATC TGA TCT GG -3′; reverse primer 5′- CTG CGA AAT GTT CAT ACT GTT G -3′). When mated with a transgenic strain expressing the tetracycline-controlled transactivator (tTA) under the regulation of the Mammary Tumor Virus promoter (MMTV-tTA) [34], three of the six founder lines (50%) showed a tTA-mediated activation of the transgene and were maintained for long-term tumor studies. One of the TetO-Tsg101 lines (founder line #1), which exhibited the highest rate of tumor onset within less than one year of latency, was chosen for detailed molecular and functional studies. This strain is listed at the Mouse Genome Informatics (MGI) registry as Tg(tetO-Tsg101,-luc)1Kuw (MGI:7643530). Further information and requests for this mouse model should be directed to the Lead Contact, Kay-Uwe Wagner.

### Other mouse strains

The generation and use of the MMTV-tTA [Tg(MMTV-tTA)25754Kuw] transgenic line to express TetO-driven responder transgenes in the mammary gland were described in our previous publications [34–36]. The presence of the MMTV-tTA transgene was determined by PCR amplification of a 364 bp fragment using a forward primer that binds to the MMTV-LTR (5′- AGT GAT AGA GCT CTT GCC TAG C -3′) and a reverse primer that binds to the tTA coding sequence (5′- GCC AAT ACA GTG TAG GCT GC -3′). TetO-H2B-GFP [Tg(tetO-HIST1H2BJ/GFP)47Efu/J] reporter strain [37] was obtained from the Jackson Laboratory. The PCR genotyping of these mice was conducted using a primer pair that amplifies a fragment of approximately 209 bps across the fusion sequence between the human histone 2B (H2B) and the enhanced green fluorescent protein (GFP) (5′- TAC AAC AAG CGC TCG ACC ATC AC -3′ and 5′- CCG TCC AGC TCG ACC AGG ATG G -3′). Detailed information about the creation and characterization of the MMTV-Flp transgenic line [Tg(MMTV-Flp)29542Kuw] can be found in previous publications by our laboratory [36, 38]. The *Rosa26*^*CAG−FSF−GFP*^ knockin reporter strain [Gt(ROSA)26Sor^tm1.2(CAG−EGFP)Fsh^] was acquired from the Jackson Laboratory (Stock No: 32038-JAX) [39]. All transgenes were carried in the FVB/N genetic background. For orthotopic transplantation experiments into immunodeficient female recipients, mammary tumor fragments of approximately 1 mm^3^ in size were implanted into the #4 inguinal mammary glands of 8–12 weeks-old athymic nude (NCr strain, Charles River) or NRG mice (NOD.Cg-Rag1tm1Mom Il2rgtm1Wjl/SzJ; RRID:IMSR_JAX:00779). Animals were housed under pathogen-free conditions in micro-isolator cages on a 12/12-h light/dark cycle. This work was conducted in accordance with the recommendations in the Guide for the Care and Use of Laboratory Animals of the National Institutes of Health. We have complied with all relevant ethical regulations. The animal study protocols were approved by the Institutional Animal Care and Use Committee of the Nebraska Medical Center and Wayne State University.

### Tumor size measurement, administration of doxycycline, and in vivo bioluminescence imaging

Mammary tumors were measured using a caliper, and tumor volumes (mm^3^) were calculated using the following equation: length (mm) × width^2^ (mm^2^)/2. The experimental endpoints to assess cancer growth in transplanted recipients and in genetically engineered mice were determined by the size of the tumor. The maximum allowed tumor size was approximately 1.5 cm in diameter, as mandated by the Institutional Animal Care and Use Committees. To suppress the MMTV-tTA-mediated expression of the TetO-Tsg101 transgene, mice were given freshly prepared Dox (doxycycline hyclate; Sigma cat.# D9891) in drinking water (2 mg/ml supplemented with 50 mg/ml sucrose). The activity of the luciferase reporter was monitored using in vivo bioluminescence imaging (IVIS200 and Bruker In Vivo Xtreme). 150 mg/kg luciferin (D-luciferin potassium salt in 0.9% saline; Caliper Life Sciences cat.# 119,222) was injected intraperitoneally ten minutes prior to the imaging procedure. During the acquisition of the images, the mice were kept under anesthesia (isoflurane) on the heated stage of the instrument. The Living Image® software was used to measure the total flux of photons emitted from each animal (photons per second).

### Histologic analysis and immunostaining

Mammary gland wholemounts were prepared by spreading the #4 inguinal mammary glands on glass slides. The tissues were fixed for 5 h in Carnoy’s solution, rehydrated, stained with Carmine Alum for several days, dehydrated, and mounted. For the routine histologic examination of mammary gland tissues and tumor specimens were fixed overnight at room temperature in 10% buffered formalin (Fisher Scientific Company) and stored in 70% ethanol before paraffin embedding, sectioning, and staining with Hematoxylin and Eosin (H&E). To examine the presence of lung metastases or expression of the H2B-GFP reporter, fresh tissue samples were spread on glass slides and examined with a Discovery.V8 fluorescence stereoscope (Carl Zeiss, Inc.). An updated protocol for immunohistochemistry or immunofluorescent staining on paraffin-embedded specimens was described by Raedler et al. [38]. Briefly, histologic sections of 5 µm were deparaffinized three times in Histo-Clear, rehydrated in decreasing concentrations of ethanol, and washed three times in 1 × PBS. Tissue sections were pressure cooked in ImmunoRetriever with Citrate (Bio SB) using a Bio SB TintoRetriever pressure cooker (116–121 °C for 4 min). After the slides cooled down to room temperature, they were rinsed in 1 × PBS and blocked with 3% bovine serum albumin (BSA) for 1 h. Next, the slides were incubated with primary antibodies overnight at 4 °C in a moisture chamber. The next day, slides were washed three times with 1 × PBS and incubated with a fluorophore-conjugated secondary antibody for 1 h in a light-protected moist chamber. After washing the slides twice with distilled water, Vectashield DAPI mounting media (Vector, H-1200) and coverslips were applied. A list of primary and secondary antibodies that were used for immunofluorescent staining is provided in *Supplementary Table* 1. Stained slides were examined with an Axio Imager microscope (Carl Zeiss, Inc.) equipped with a SPOT FLEX camera (Diagnostic Instruments, Inc.).

### Activation of the TetO-Tsg101 transgene in embryonic fibroblast cell cultures

Similar to the cloning of the pBabe-tTA [40], a pBabe-rtTA-puro retroviral expression vector that expresses the reverse tetracycline-inducible transactivator was generated by inserting a blunted *Eco*RI/*Bam*HI fragment of the rtTA coding sequence with an N-terminal nuclear localization signal (NLS) from pUHD172-1 into the blunted *Eco*RI site of the pBabe-puro plasmid. To derive mouse embryonic fibroblasts (MEFs) from 12.5-day-old embryos, wild-type FVB females were mated with TetO-Tsg101 transgenic males. MEFs were explanted and maintained in Dulbecco’s modified Eagle’s medium (DMEM) that was supplemented with 10% fetal bovine serum, 2 mM glutamine, 0.1 mM nonessential amino acids, 10 µg/ml gentamycin, 100 units/ml penicillin, and 100 µg/ml streptomycin. After genotyping, primary MEFs that carried the TetO-Tsg101 transgene were immortalized according to the standard 3T3 protocol. To activate the TetO-Tsg101 transgene, MEFs were plated at a density of 3–4 × 10^5^ cells per 10-cm culture dish and infected with pBabe-rtTA-puro retroviral particles. As a control, isogenic MEFs were infected with the original pBabe-puro retrovirus. Forty-eight hours after infection, cells were selected in complete medium containing 7 µg/ml puromycin. To start the rtTA-mediated transactivation, puromycin-resistant cells were maintained for 24–96 h in the complete DMEM medium that contained 1 µg/µl Dox. For extended time points, the cell media were changed after 48 h. To monitor the activation of the luciferase reporter, luciferin was added to the cell culture media (150 μg/ml) three minutes prior to the acquisition of bioluminescent images using the IVIS200. Cells were washed in 1 × PBS and collected for immunoblotting after 48 and 96 h of treatment with Dox as well as 24 and 48 h after Dox withdrawal.

### Human breast cancer cell lines, immunoblot analyses

The acquisition and maintenance of MCF 10A and 12A mammary epithelial cells as well as the human breast cancer cell lines used in this study were described in our previous publication [41]. Human cell pellets and homogenized mouse tissues were sonicated in complete lysis buffer containing 1% Nonidet P-40, 0.5% sodium deoxycholate, 0.1% SDS, 1 mM phenylmethylsulfonyl fluoride (PMSF), 0.4 units/ml aprotinin, 1 mM NaF, leupeptin, and 0.1 mM sodium orthovanadate and kept on ice for 30 min. Extracts were resolved by SDS-PAGE and blotted onto polyvinylidene fluoride (PVDF) membranes. After blocking the membranes for 1 h in 5% dry milk or 5% BSA (phosphotyrosine-specific antibodies) in 1 × Tris-buffered saline with 0.05% Tween-20 (TBST) buffer, the membranes were incubated with primary antibodies in the blocking buffer at 4 °C overnight. A list of primary antibodies for immunoblotting as well as recommended dilutions are provided in *Supplementary Table* 2. Subsequently, the membranes were washed in 1 × TBST and incubated for 1 h with one of the corresponding horseradish peroxidase-conjugated secondary antibodies listed in *Supplementary Table* 2. After three washes with 1 × TBST, two washes in 1 × TBS (Tris-buffered saline without Tween 20) and finally in ultrapure water, the protein bands were detected using the ECL chemiluminescence kit for Western blot analysis [KwikQuant Ultra Digital-ECLTM Substrate Solution (cat.# R1002)] according to the instructions by the manufacturer (Kindle Biosciences, LCC). Chemiluminescence and brightfield images of the blots with size markers were taken using a D1001 KwikQuant Imager (Kindle Biosciences, LLC). Membranes were stripped using a mild glycine stripping buffer (Abcam protocol) for consecutive detection of various proteins. For the analysis of TSG101 expression in human breast cancer cell lines, we used Ultra Blue films (VWR) for the chemiluminescence detection. The films were scanned and protein bands were quantified using the ImageJ software. The intensity of the TSG101 protein bands was normalized to the beta-actin loading control, and the resulting TSG101 expression values of individual cancer cell lines were plotted as fold change in comparison to the average TSG101 expression in the normal cell lines (set as 1).

### RNA-Sequencing, gene set enrichment, and cluster analyses

Total RNA was extracted from flash-frozen mammary tumors and normal mammary gland tissues using the RNeasy Mini Kit (QIAGEN). The concentration of the RNA was determined on a NanoDrop spectrophotometer, and the integrity of the RNA was validated using gel electrophoresis. The RNA expression library construction and next-generation sequencing were performed at the Genomics Core Facility at UNMC (NextSeq 500). The quality of sequenced reads was determined using FastQC (v0.11.9; http://www.bioinformatics.babraham.ac.uk/projects/fastqc). For GSEA, the ranked gene list was derived from a differential expression analysis between FVB mammary glands and TSG101-overexpressing mammary tumors. The genes were ranked by fold change and false discovery rate (FDR). For the differential expression analysis, the 75 base pair paired-end reads were mapped to the mm10 mouse reference genome with Rsubread (v2.0.0) [42]. Transcript abundance was determined using featureCounts from the Rsubread package. Low abundance transcripts (cpm ≤ 5) that occurred in more than half of the samples were excluded from the subsequent analyses. The R package edgeR (v3.28.0) [43] was used to normalize the transcript counts and to perform differential expression analysis. Transcript abundance was estimated in counts per million (cpm). Genes that showed differential expression by more than twofold and an FDR (False Discovery Rate) of below 0.05 between the sample groups were considered as significantly deregulated. The gene set enrichment analysis was performed with R-function gseKEGG [44]. The corresponding heatmaps were created by log2 transforming the count data, extracting the significantly deregulated genes that belong to the corresponding pathways, and z-score transformation of the count data by gene. Heatmaps were plotted with the R-function heatmap.2 (package: gplots v3.0.3) (http://cran.r-project.org/web/packages/gplots/index.html).

To perform the unsupervised hierarchical cluster analysis of mammary tumors from MMTV-tTA TetO-Tsg101 females together with reference tumors from 31 diverse mouse mammary cancer models and normal mammary glands from FVB and BALB/c mice, RNA-sequencing (RNA-seq) data was upper quartile normalized and combined with previously published RNA-seq data (GSE124821, GSE223630, and GSE118164) [45–47]. Samples were subset with the 1,723 murine intrinsic genes, log2 transformed, and median-centered prior to centroid linkage clustering in Cluster 3.0 (v3.0) [48]. The data was visualized through Java TreeView (Version 1.2.0 for Mac) [49].

### Statistical analysis

All graphic illustrations and statistics were performed with GraphPad Prism 6 software (GraphPad Software, Inc., La Jolla, CA). All reported data points and measurements were taken from individual animals and distinct samples. The tumor-free survival distributions between animals were assessed using the log-rank test. P values < 0.05 ( ∗), < 0.01 (∗ ∗) or < 0.001 (∗ ∗ ∗) were considered statistically significant. RNA sequencing analysis-based statistical methods are described in the documentation of the respective R-packages and their corresponding manuscripts. The default statistical parameters were used for the differential gene expression analysis and the gene set enrichment analysis.

## Results

### The TSG101 protein is upregulated in human breast cancer cells

**Fig. 1:**
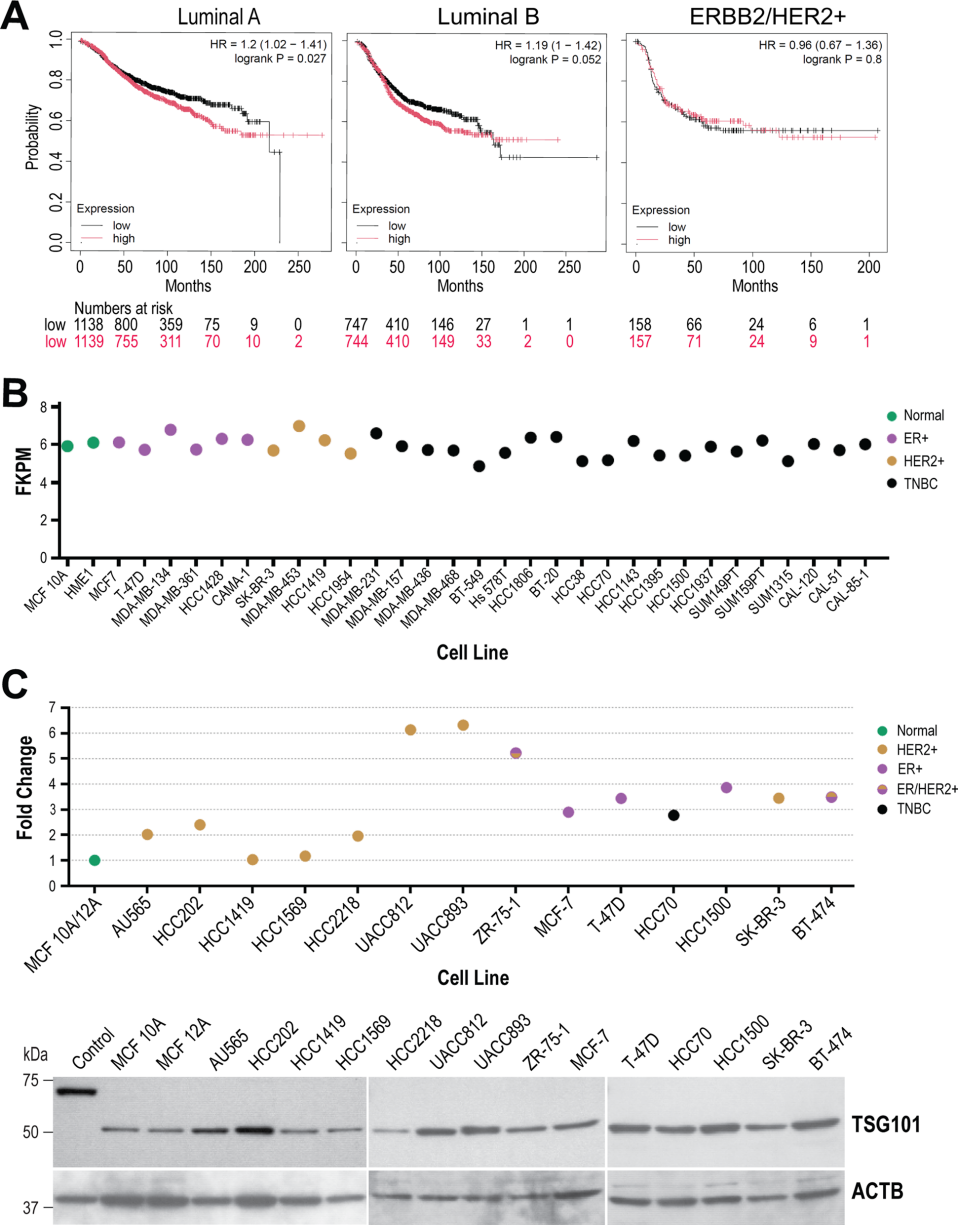
Human breast cancer cell lines exhibit an upregulated expression of the TSG101 protein. **A**. Kaplan–Meier survival curves assessing the correlation between *TSG101* mRNA expression levels and survival of patients with luminal-type and Her2-positive breast cancers (source: https://kmplot.com/). **B**. Normalized gene expression levels of *TSG101* transcripts (Fragments Per Kilobase of transcript per Million mapped reads, FPKM) in 2 normal (MCF10A, HME1) and 30 human breast cancer cell lines from the Harvard Breast Cancer Profiling Project (ID:20,348). The cell lines and their FKPM values were arranged and color-coded according to the major immune-histological breast cancer subtypes they represent. **C**. Immunoblot analysis of TSG101 on two normal and 14 breast cancer cell lines. Cell lysates from TSG101 knockout mouse embryonic fibroblasts that express a CLIP-tagged human TSG101 protein served as a control to validate the specificity of the antibody. Beta-actin (ACTB) served as a loading control. The intensity of the TSG101 protein bands was normalized to the loading control, and the resulting TSG101 expression values of individual cancer cell lines were plotted as fold change in comparison to the average TSG101 expression in the normal cell lines (set as 1). The plotted TSG101 values of the cell lines are color-coded according to the major immune-histological breast cancer subtypes

Using immunohistochemistry on formalin-fixed sections of 16 human breast cancers and 16 normal breast tissues and benign hyperplasia, we found that the levels of TSG101 were significantly higher in approximately half of the invasive breast cancer cases [20]. Recurrent mutations in the gene encoding TSG101 have not been identified in human breast cancers, but elevated levels of *TSG101* transcripts are associated with a more dismal prognosis for luminal-type breast cancers (Fig. 1A). Interestingly, the analysis of RNA sequencing data of 2 normal and 30 human breast cancer cell lines from the Harvard Breast Cancer Profiling Project (ID:20,348) shows that the expression of the *TSG101* transcript is nearly identical between normal and malignant cancer cells that represent the three major immune-histological tumor subtypes (Fig. 1B). In contrast to the mRNA expression, we observed that the TSG101 protein expression was 2–sixfold higher in 10 of 14 breast cancer cell lines in comparison to two normal cell lines that we examined by immunoblot analysis (Fig. 1C). The specificity of the antibody to recognize the human TSG101 protein (hTSG101) was validated using cell lysates from conditional knockout mouse embryonic fibroblasts that lack both alleles of the endogenous gene (*Tsg101*^*−/−*^) and express a CLIP-tagged or a dTomato reporter fusion protein of hTSG101. A direct comparison of selected cell lines of the major breast cancer subtypes that are included in the transcriptomic data set and in the immunoblot (e.g., MCF-7, T-47D, SK-BR-3, HCC70) clearly shows that the levels of the proteins deviate significantly from minor variations in the mRNA expression, suggesting that altered posttranslational mechanisms contribute to the functionality of this oncoprotein. Moreover, none of the untransformed mammary epithelial cells and breast cancer cell lines exhibited shorter TSG101 protein variants that may have originated from aberrant or alternative mRNA splice products.

### Overexpression of TSG101 in the mammary gland epithelium causes ductal hyperplasia and mammary cancer

**Fig. 2:**
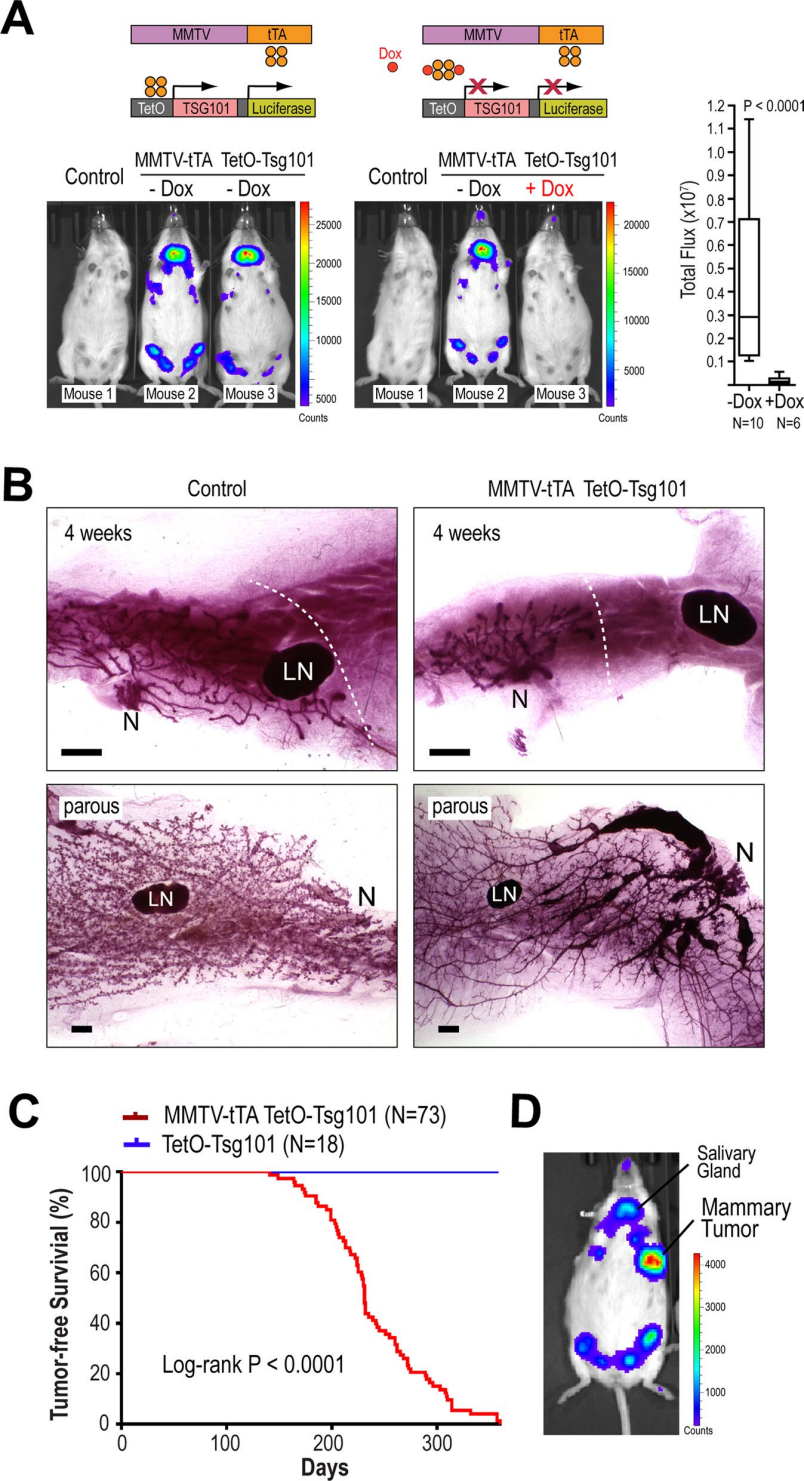
Conditional expression of TSG101 under the control of the tetracycline-controlled transactivator (tTA) in the mammary gland causes ductal hyperplasia and mammary tumors. **A**. In vivo bioluminescence imaging (IVIS200) of two MMTV-tTA TetO-Tsg101 double transgenic females (mouse 2, 3) before and after one of the two experimental animals (mouse 3) was given doxycycline (Dox) for 48 h. A TetO-Tsg101 single transgenic female (mouse 1) served as a control. The schematic of the double transgenic model illustrates the functionality of the Dox-controlled expression system as well as the co-expression of the luciferase reporter from the bicistronic TetO-Tsg101 transgene. The results of the quantitative bioluminescent image analysis of 10 untreated and 6 Dox-treated mice are shown as a box plot. **B**. Carmine Alum-stained wholemounts of mammary glands from nulliparous (4 weeks, upper panels) and nonpregnant multiparous (lower panels) double transgenic females and TetO-Tsg101 single transgenic controls; LN, lymph node; N, nipple region; bars, 1 mm. The dotted lines mark the leading boundaries of extending ducts from the nipple towards the lymph nodes and epithelium-free mammary fat pads at 4 weeks of age. **C**. Kaplan–Meier survival plot of multiparous MMTV-tTA TetO-Tsg101 females and multiparous TetO-Tsg101 single transgenic controls. Statistical significance in tumor-free survival between the two experimental groups was calculated with the log-rank test, *P* < 0.0001. **D**. In vivo bioluminescence imaging to validate the MMTV-tTA-mediated expression of the TetO-Tsg101 transgene in a palpable mammary tumor

To study whether the overexpression of TSG101 is sufficient to initiate the formation of mammary cancer and to assess whether this oncoprotein is required for tumor maintenance, we generated transgenic mice that permit a tissue-specific and temporally controlled overexpression of TSG101 in the mammary epithelium. We cloned a bicistronic transgene [TetO-Tsg101-IRES-luciferase] where, upon binding of the tetracycline-controlled transactivator (tTA) to its operator/promoter (TetO), the full-length mouse TSG101 is expressed along with a luciferase reporter (Suppl. Fig. S1A). Following pronuclear injection, we obtained six founder animals that transmitted the TetO-Tsg101 transgene to their progeny. To test the transactivator-dependent expression of the transgene, we crossed the TetO-Tsg101 founder lines with transgenic mice expressing the tTA (Tet-Off repressor) under the control of the Mouse Mammary Tumor Virus promoter (MMTV-tTA) [34]. Using in vivo bioluminescence imaging, we determined that three of the six founder lines (1, 6, and 11) exhibited the anticipated expression of luciferase in the mammary and salivary glands of MMTV-tTA TetO-Tsg101 double transgenic females (Fig. 2A). The TetO-Tsg101 transgene was silenced in double transgenic females after administration of doxycycline (Dox) in drinking water for 48–72 h as determined by quantitative bioluminescent imaging on 10 untreated and 6 Dox-treated mice (Fig. 2A). To confirm the expression of the TSG101 protein from the bicistronic transgene, we cultured primary mouse embryonic fibroblasts (MEFs) from founder line #1 and infected them with a retroviral vector expressing the reverse tetracycline-inducible transactivator (rtTA, Tet-On). Following the administration of Dox, we validated the transgene activation using bioluminescence imaging and by immunoblot analysis of the transgenic TSG101 protein which carries a hemagglutinin (HA) tag on its N-terminus (Suppl. Fig. S1B–C). While the expression of luciferase and exogenous TSG101 from the TetO-Tsg101 transgene were dependent on the rtTA and administration of Dox, the total levels of TSG101 remained relatively stable in cultured fibroblasts. Similar to the phenomenon first reported by Feng et al. [30], we observed a downregulation of the endogenous TSG101 protein following a short-term upregulation of the transgenic protein (Suppl. Fig. S1C, upper panel). This suggested that the normal autoregulatory feedback mechanism controlling the TSG101 protein level was not perturbed in fibroblasts that expressed the TetO-Tsg101 transgene under the control of a retroviral transactivator. The withdrawal of Dox for 24–48 h resulted in a swift suppression of the rtTA-mediated activation of the TetO-Tsg101 transgene (Suppl. Fig. S1C, lower panel).

While the Dox-inducible upregulation of exogenous TSG101 had no noticeable effect on the growth of cultured fibroblasts, we observed an initial delay in the pre-pubertal development of mammary glands in transgenic mice overexpressing TSG101. The MMTV-tTA is already highly expressed in the rudimentary mammary epithelium of newborn animals where it transactivates TetO-driven responder transgenes in the absence of Dox (Tet-Off) [34]. At four weeks of age, the elongation of mammary ducts in the inguinal #4 glands of MMTV-tTA TetO-Tsg101 females was stunted (Fig. 2B, upper panel), but the protracted development of the ductal tree in post-pubertal double transgenic mice became indistinguishable from littermate controls after eight weeks of age (Suppl. Fig. S2A, upper panel). In contrast to the inhibitory effect on the growth of the main collecting ducts, the overexpression of TSG101 during lactation did not affect the proliferation and functional differentiation of secretory alveolar cells and MMTV-tTA TetO-Tsg101 dams were able to lactate normally (Suppl. Fig. S2A, B). However, the high expression of TSG101 during pregnancy and lactation caused extensive and, in some cases, very severe forms of ductal hyperplasia near the nipple areas in the thoracic and inguinal mammary glands of involuted multiparous females (Fig. 2B, lower panel, Suppl. Fig. S2C, upper panel). While mammary cancer formation was not observed in single transgenic TetO-Tsg101 or MMTV-tTA control animals, double transgenic females of the three expressing founder lines developed palpable mammary tumors by 18 months of age. The detailed histological and molecular analysis of mammary cancers was carried out on tissue specimens of founder line #1, which showed a very consistent rate of tumor formation with a median latency of 231 days (N = 73)(Fig. 2C). Palpable tumors that originated in any of the ten mammary glands showed a strong expression of luciferase, indicating that the MMTV-tTA-driven TetO-Tsg101 transgene was still highly active in progressing mammary cancers (Fig. 2D). Although less severe forms of ductal hyperplasia were present in the main collecting ducts of nulliparous MMTV-tTA-driven TetO-Tsg101 females (Suppl. Fig. S2C, lower panel), the formation of palpable mammary tumors was dependent on parity and only 2 of 14 nulliparous females that were monitored for 12–18 months developed mammary carcinomas at 214 and 362 days-of-age.

### TSG101-induced mammary tumors are adenosquamous carcinomas and adenomyoepitheliomas

**Fig. 3:**
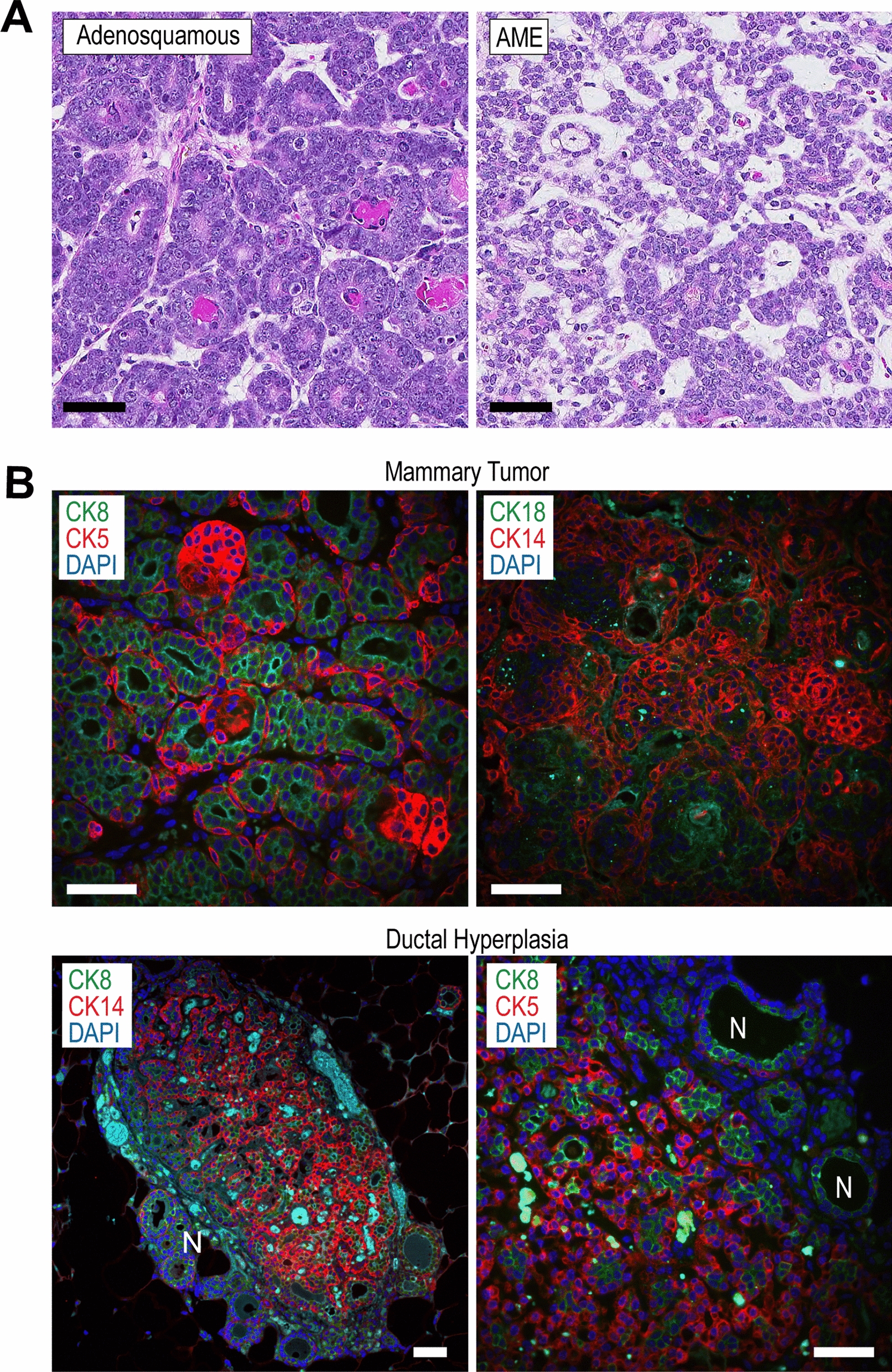
TSG101 overexpression causes the development of adenosquamous carcinomas with regional areas of AMEs that are comprised of transforming luminal and basal epithelial cells. **A**. Hematoxylin and eosin (H&E)-stained histologic sections of mammary tumors from MMTV-tTA TetO-Tsg101 double transgenic females; bars, 50 µm. **B**. Immunofluorescent staining of luminal epithelial cytokeratins 8 and 18 (CK8, CK18) as well as basal epithelial cytokeratins 5 and 14 (CK5, CK14) in adenosquamous carcinomas (upper panels) and ductal hyperplasias (lower panels); N, adjacent normal epithelial ducts, bars, 50 µm. Slides were counterstained with DAPI

The histopathologic analysis revealed that large tumors overexpressing TSG101 were adenosquamous mammary carcinomas. Some of these tumors contained areas that exhibited typical characteristics of adenomyoepitheliomas (AMEs) that were more predominant in the hyperplastic ducts (Fig. 3A; Suppl. Fig. S3A). Similar to neoplasms of the same histopathologic subtypes in humans, all TSG101-induced mammary tumors were comprised of cytokeratin 8 and 18 (CK8/18)-positive luminal epithelial cells and basal cells expressing CK5 and CK14 (Fig. 3B, upper). Interestingly, luminal and basal epithelial cells were already present within hyperplasia of the main collecting ducts (Fig. 3B, lower), suggesting that both epithelial lineages were coevolving during the subsequent stages of mammary tumorigenesis. The TSG101-induced mammary cancers were slow-growing, and regardless of their final dimensions at the time of necropsy (1–2 cm), only 2% of the tumor-bearing mice had metastatic lesions in their lungs as determined by stereoscopic examination (Suppl. Fig. S3B). This suggested that similar to the corresponding human neoplasms, adenosquamous mammary carcinomas in TSG101-overexpressing females were typically low-grade. This notion is supported by the fact that transplanted tumor fragments remained dormant and did not give rise to secondary large mammary cancers after 4–5 months when orthotopically transplanted into athymic nude (N = 16) and NRG (N = 16) immunocompromised recipient females (Suppl. Fig. S3C). Also, numerous attempts to establish primary and immortalized TSG101-overexpressing tumor cell lines were unsuccessful likely due to the silencing of the MMTV-tTA and TetO-Tsg101 transgene expression in cultured tumor cells as determined by bioluminescence imaging (not shown). Consequently, all subsequent biochemical and molecular studies were conducted on primary tumor specimens.

### Triple-negative tumors overexpressing TSG101 exhibit high PI3K/AKT and JAK/STAT signaling

**Fig. 4:**
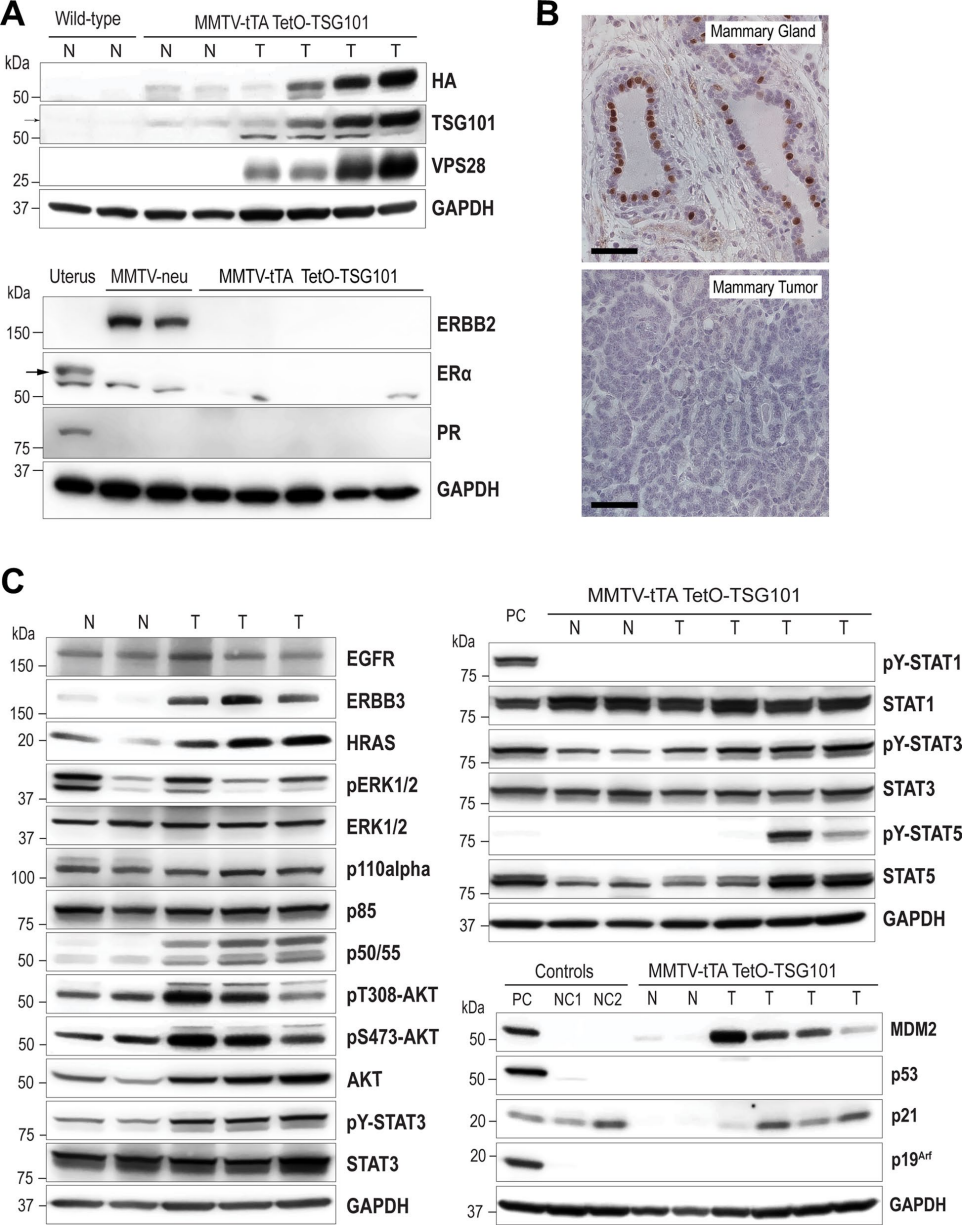
TSG101-induced mammary tumors are triple-negative and exhibit a robust activation of PI3K/AKT and JAK/STAT signaling. **A**. Immunoblot analysis of HA-tagged TSG101 and its binding partner VPS28 as well as the estrogen and progesterone receptors (ERα, PR) and the epidermal growth factor receptor 2 (ERBB2/HER2) in mammary tumors (T) and normal mammary gland tissues (N) of parous MMTV-tTA TetO-Tsg101 double transgenic females; tissue specimens of normal mammary glands (N) and uterus of wild-type FVB females as well as ERBB2-overexpressing mammary tumors (MMTV-neu) served as controls. **B**. Immunohistochemical staining of nuclear FOXA1 in normal mammary gland and TSG101-induced tumor tissues, bars, 50 µm. **C**. Western blot analysis of selected receptor tyrosine kinases and downstream MAP3K and PI3K/AKT signaling effectors (left), as well as STAT proteins and cell cycle regulators (right); PC, positive controls, mutant KRAS-induced mouse mammary tumors expressing tyrosine phosphorylated STAT1/3 and high levels of mutant p53 and p19^Arf^; NC, negative controls, NC1 p19^Arf^-deficient NIH3T3 cells; NC2, mouse embryonic fibroblasts (MEFs) with a targeted double knockout of MDM2 and p53. GAPDH was used as loading controls in panels **A** and **C**

Compared to the normal mammary glands of MMTV-tTA TetO-Tsg101 double transgenic females, the expression of exogenous TSG101 was significantly higher in most mammary cancers (Fig. 4A). This may suggest that the initiation of tumorigenesis is accompanied by the loss of the autoinhibitory feedback mechanism that constrains a deregulated expression of the TSG101 protein. Similar to the reported posttranslational downregulation of the ESCRT-1 binding partner VPS28 in siRNA-mediated TSG101 knockdown cell lines [50, 51], we observed a concomitant upregulation of VPS28 in mammary tumors with high overexpression of TSG101. All adenosquamous carcinomas in the TSG101-induced mammary tumor model lacked expression of the estrogen and progesterone receptors as well as amplification of ERBB2. The absence of FOXA1 staining in these carcinoma cells supported the finding that the growth of the triple-negative TSG101-induced mammary tumor cells was likely not controlled by steroid hormones (Fig. 4B).

Adenosquamous and adenofibromatous mammary tumors often result from the deregulated activation of the RAS and PI3 kinase signaling pathways [52, 53]. Compared to adjacent normal mammary glands, TSG101-overexpressing carcinomas show higher levels of ERBB3, HRAS, and active AKT (Fig. 4C, left). The upregulation of the p50/p55 regulatory subunits of the PI3 kinase in tumor cells is a consequence of an increase in inflammatory cytokine signaling through the JAK/STAT pathway, in particular tyrosine-phosphorylated (i.e., activated) STAT3 (Fig. 4C, right). A subset of tumors exhibited a persistent activation of STAT5, which indicates the presence of a significant fraction of differentiated luminal epithelial cells. Despite the broad activation of JAK/STAT signaling cascades and low-grade histopathological features of these adenosquamous carcinomas, the TSG101-induced tumors lacked active STAT1, which is a suggested tumor suppressor. Interestingly, the overexpression of TSG101 led to a significant increase in MDM2 expression in established mammary tumors (Fig. 4C, right). The concurrent upregulation of p21, along with a sustained low expression of p19^Arf^ as well as the absence of high p53 protein levels were indicative that the genesis of triple-negative adenosquamous carcinomas was not associated with mutations in *Trp53*.

### The gene expression profiles of TSG101-overexpressing tumors are similar to mammary cancers that originate late in MMTV-Wnt1 transgenic females

**Fig. 5:**
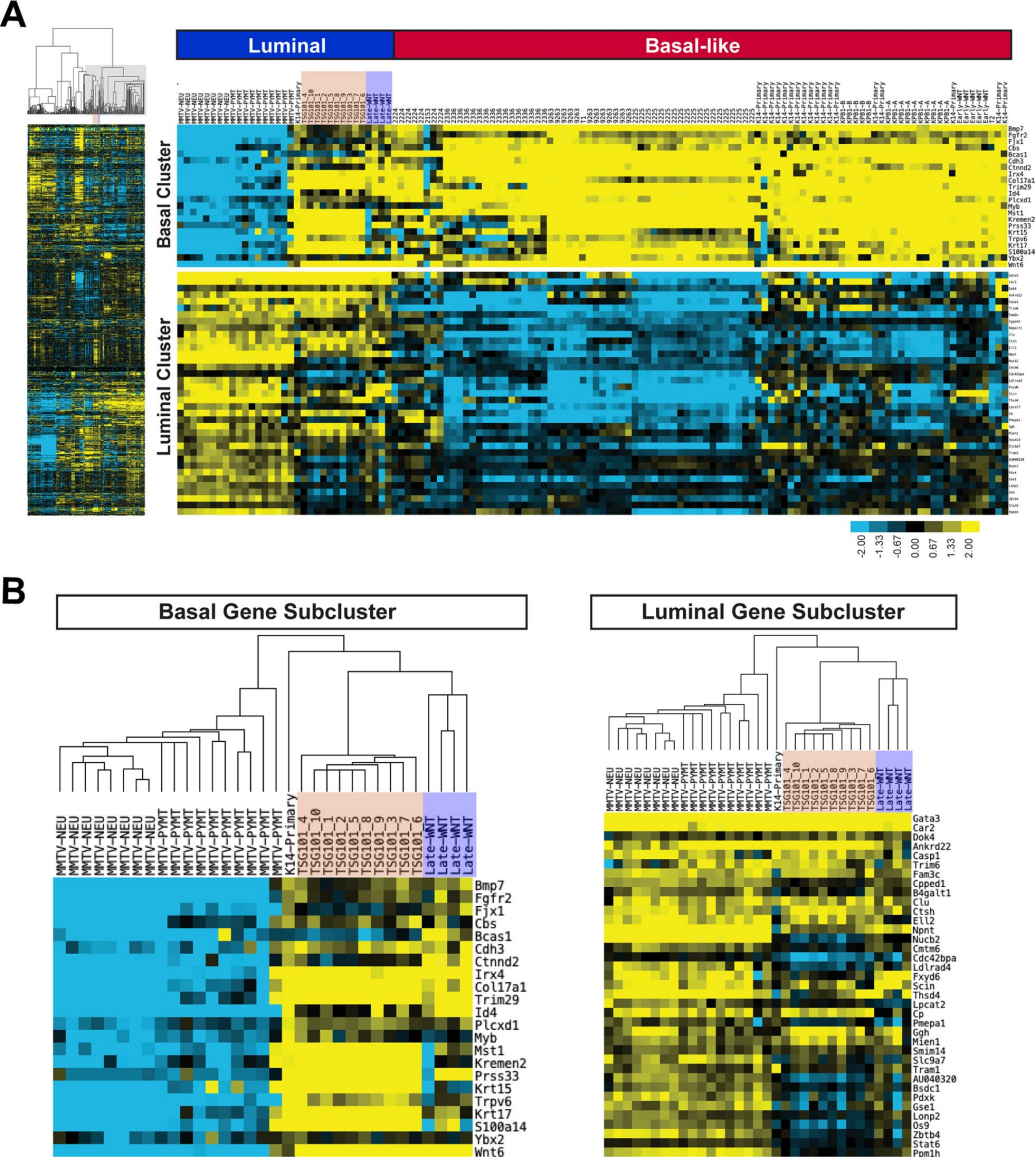
Unsupervised hierarchical clustering of TSG101 overexpressing mammary tumors with mouse reference tumors and normal mammary glands. **A**. Left, overview of the complete heatmap resulting from the unsupervised hierarchical clustering of the expression of 1,723 intrinsic genes among 251 individual mammary cancers and normal mammary glands, representing 31 reference tumor models, including cancer tissues that originated late in MMTV-Wnt1 transgenic females (light blue; Wnt1-Late.^Ex^; N = 4;) and that clustered together with tumors from TSG101-overexpressing females (light red; MMTV-tTA TetO-Tsg101; N = 10). Right, a selected subset of mammary cancer models from the entire cluster showing that tumors from TSG101-overexpressing females share expression patterns with basal-like and luminal mammary tumors. **B**. Basal and luminal gene expression subclusters showing similarities between mammary tumors that are overexpressing TSG101 and Wnt1

We performed RNA sequencing on ten tumor specimens to classify TSG101-overexpressing mammary carcinomas based on their intrinsic gene expression profiles. The results of the unsupervised hierarchical clustering based on 1,723 intrinsic genes of these tumors alongside 251 tissue samples representing 31 reference mammary tumor models and normal mammary glands from two genetic backgrounds (i.e., FVB and BALB/c) are shown in Suppl. Fig. S4. The computational analysis revealed that TSG101-overexpressing tumors are most similar to luminal-type mammary cancers that developed late in MMTV-Wnt1 (Wnt1-Late^Ex^) transgenic females (Fig. 5A). A shared feature of the TSG101 and late Wnt1-induced luminal-type mammary tumors is the expression of genes that belong to the basal gene cluster (Fig. 5B, left). In addition, there were several genes within the luminal gene expression subcluster that discriminated TSG101-overexpressing and late Wnt1-induced mammary cancers from typical luminal tumors that originated in MMTV-neu and MMTV-PyMT mice (Fig. 5B, right). TSG101-overexpressing tumors were relatively slow-growing, which is also evident from the reduced expression of genes within the proliferation subcluster (P, black) shown in Suppl. Fig. S4.

To gain insight into the molecular pathways that are associated with TSG101-induced mammary tumorigenesis, we compared the gene expression profiles of TSG101-induced mammary tumors with RNA sequencing data from mammary gland tissues of three FVB wild-type females. Gene set enrichment analysis (GSEA) confirmed the deregulated expression of genes associated with Wnt signaling as well as cytokine receptor and JAK/STAT pathway activation (Suppl. Fig. S5). Several differentially regulated genes within these pathways are key signaling nodes and effectors that control biological processes, such as the pluripotency of stem and progenitor cells and the genesis of basal cell carcinoma. Another group of deregulated genes in TSG101-overexpressing tumors is functionally linked to microRNAs (Suppl. Fig. S5). These genes play a role in several of the proposed multifaceted functions of TSG101, including the biogenesis of exosomes and the release of local growth factors such as inflammatory cytokines and Wnts [54, 55].

### Overexpression of TSG101 in luminal progenitors promotes cellular heterogeneity

**Fig. 6:**
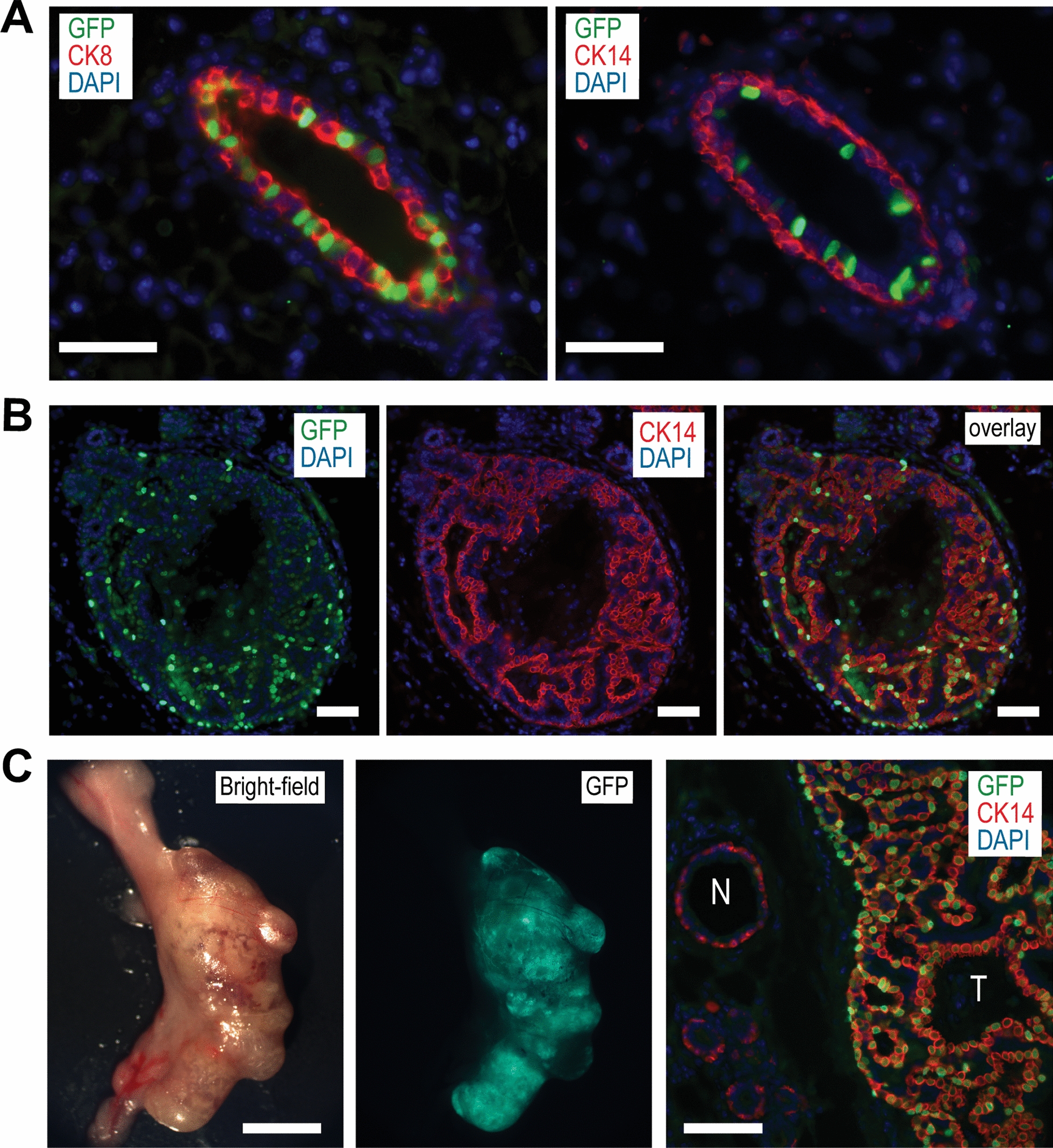
Basal-like tumor cells in heterogeneous mammary carcinomas overexpressing TSG101 originate from luminal epithelial cells. **A**. Immunofluorescent (IF) co-staining of nuclear GFP along with luminal cytokeratin 8 (CK8) or basal cytokeratin 14 (CK14) in the mammary gland of MMTV-tTA TetO-H2B-GFP double transgenic females; bars 50 µm. Note that without the expression of an oncogene, the MMTV-tTA transactivates TetO-driven responder transgenes in a subset of luminal epithelial cells. **B**. IF staining of the MMTV-tTA-driven nuclear GFP reporter in CK14-positive basal epithelial cells in a ductal hyperplasia that developed in MMTV-tTA TetO-Tsg101 TetO-H2B-GFP triple transgenic females; bars, 50 µm. **C**. Stereoscopic bright-field image (left) with a corresponding GFP fluorescent image (middle) of a mammary tumor that developed in an MMTV-tTA TetO-Tsg101 TetO-H2B-GFP triple transgenic female; bar, 0.5 cm. The right panel shows a histologic section of the mammary tumor (T) with adjacent normal duct (N) that was co-stained for GFP and CK14; bar 50 µm. DAPI was used as a counterstain in panels **A**-**C**

In addition to their gene expression profiles, TSG101-induced mammary cancers and Wnt1-Late^Ex^ tumors share several commonalities, such as their mixed composition of cancer cells with luminal and basal epithelial cell characteristics. The observation that diverse epithelial subtypes were already present in ductal hyperplasia of TSG101-overexpressing females prior to tumor onset (Fig. 3B) provided a unique opportunity to study whether tumor cell heterogeneity is a result of potential paracrine effects of the oncogene or a luminal-basal transition of transforming epithelial cells. Using a TetO-H2B-GFP reporter transgene, we observed that in the absence of the TetO-Tsg101 transgene, the MMTV-tTA is expressed in a mosaic fashion in a subset of CK8-positive luminal epithelial cells of mammary ducts (Fig. 6A). The presence of nuclear GFP in most CK14-positive cells in hyperplastic ducts of MMTV-tTA TetO-Tsg101 TetO-H2B-GFP triple transgenic females indicated that basal cells in the heterogenous preneoplastic lesions likely originated early from luminal progenitors (Fig. 6B). Consequently, they are not groups of cells that are co-amplified though paracrine signals from oncogene-expressing luminal cells. In support of this notion, the GFP reporter was still expressed in basal epithelial cells of large adenosquamous carcinomas (Fig. 6C). These collective observations were validated in MMTV-tTA TetO-Tsg101 females that also carry the MMTV-Flp transgene in combination with the *Rosa26*^*CAG−FSF−GFP*^ reporter allele. Similar to the MMTV-tTA, the expression of the MMTV-Flp transgene is confined to the luminal epithelium where the Flp recombinase initiates a constitutive expression of GFP from the ubiquitously active *Rosa26* locus (Suppl. Fig. S6A). This dual reporter system was used to label luminal epithelial cells and their progeny during the early stages of TSG101-associated transformation in parous MMTV-tTA TetO-Tsg101 MMTV-Flp *Rosa26*^*CAG−FSF−GFP*^ females (Suppl. Fig. S6B). Although the MMTV-tTA and MMTV-Flp expression profiles are mosaic and not identical in all cells, hyperplastic ducts in quadruple transgenic mice were GFP-positive. The results from the immunofluorescent co-staining of CK14 and GFP on histologic sections of these lesions suggested that transforming epithelial cells with basal-like features likely originated from the luminal epithelium.

### Exogenous TSG101 is required for tumor progression and the survival of carcinoma cells

**Fig. 7:**
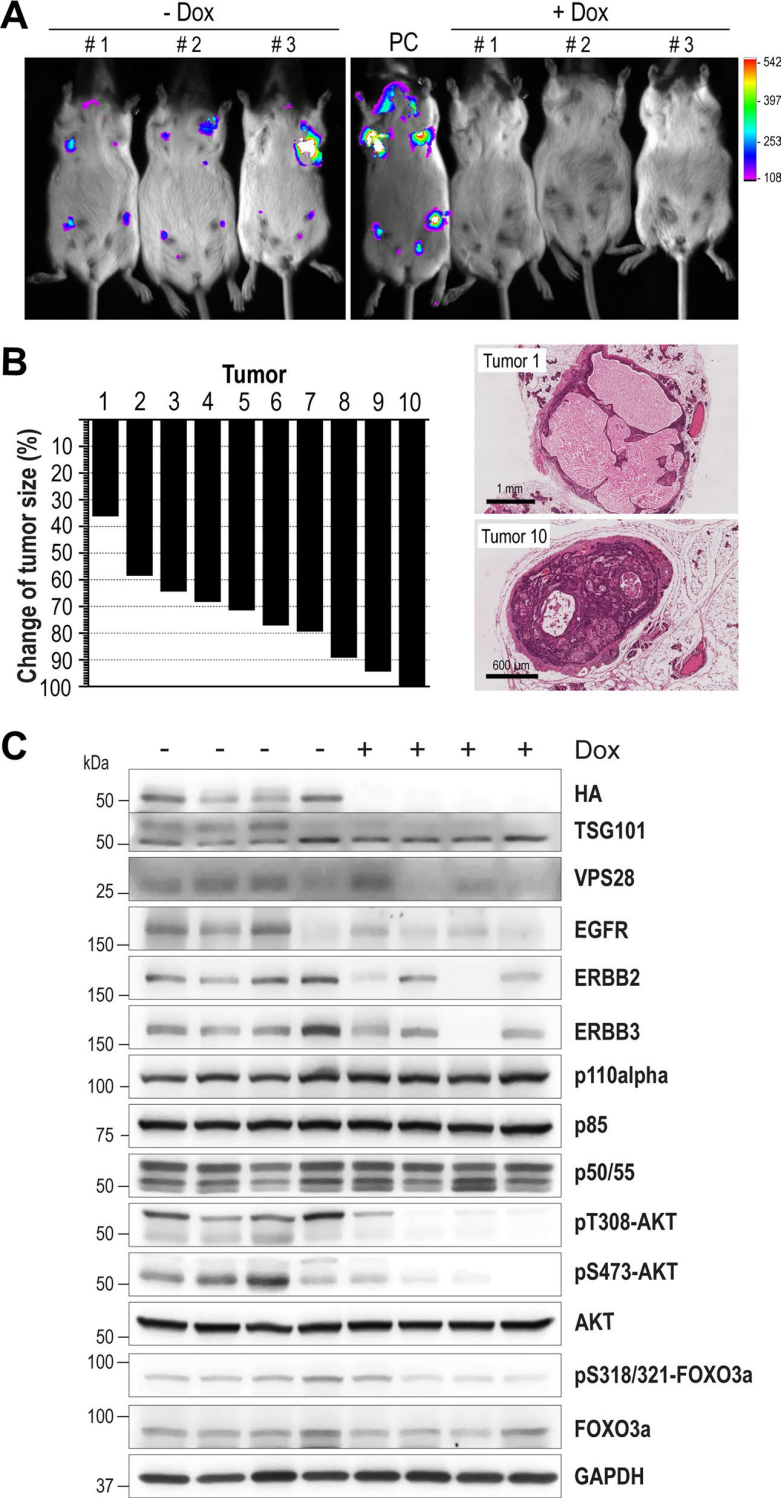
Sustained expression of exogenous TSG101 is required for the survival of mammary carcinoma cells in vivo. **A**. In vivo bioluminescence imaging (Bruker In Vivo Xtreme) of tumor-bearing MMTV-tTA TetO-Tsg101 mice before (-Dox) and after administration of doxycycline (+ Dox) in the drinking water for 72 h (N = 3); PC, positive control, tumor-bearing mouse that was not treated with Dox. **B**. Relative changes in tumor size (N = 10 animals) before and 30 days after continuous treatment with Dox. The right images are hematoxylin and eosin (H&E)-stained sections of the tumors with the least (tumor 1) and macroscopically near-complete regression (tumor 10) at the end of the treatment period; bars, 1 mm (tumor 1) and 600 µm (tumor 10). **C**. Western blot analysis of HA-tagged TSG101, ERBB receptor tyrosine kinases, and downstream mediators of PI3K/AKT signaling in mammary carcinomas before and after 72 h of treatment with Dox. GAPDH was used as a loading control

The observation that luciferase was expressed from the bicistronic TetO-Tsg101 transgene in established mammary tumors (Fig. 2C) suggested that the overexpression of the oncoprotein is continuously required for the maintenance and progression of adenosquamous carcinoma cells. To experimentally address this notion, we administered Dox in the drinking water of 10 tumor-bearing females. The ligand-mediated suppression of the TetO-Tsg101 transgene was validated by bioluminescence imaging 48 and 72 h after the first administration of Dox (Fig. 7A). Tumors were measured every other day for four weeks while the animals were continuously treated with Dox. At the end of the treatment period, 8 of the 10 tumors were more than 60% smaller in size (Fig. 7B, left). The histological examination of residual cancer tissues of Dox-treated animals showed that the size of the tumor was not an accurate readout for the effects of the ablation of exogenous TSG101 (Fig. 7B, right). The tumor with the least reduction in size (tumor 1) was cystic and filled with necrotic fluid. On the other end of the spectrum, residual carcinoma cells were still present at primary tumor sites where carcinomas exhibited a near complete macroscopic regression in response to Dox treatment (tumors 9 and 10). None of the regressed tumors showed a recurrence or increase in size while the mice were treated with Dox for a month, and Ki-67-positive cells were not detected in residual cancer cells (Suppl. Fig. S7).

Next, we collected tumor specimens from animals that were treated with Dox for 72 h to investigate the immediate impact of the downregulation of TSG101 on cytokine and receptor tyrosine kinase signaling. While the short-term treatment with Dox had no immediate effect on the STAT3-mediated expression of the PI3K subunits p50/p55, the ablation of exogenous TSG101 resulted in a notable decline in the expression of individual EGFR/ERBB receptors as well as the downstream activation of AKT and its effectors (Fig. 7C), including Cyclin D1 (Suppl. Fig. S8). We also observed a significant reduction in the expression of MDM2 and p21^Cip1^ in mammary tumors as a consequence of the downregulation of TSG101 (Suppl. Fig. S8). The collective results suggest that the growth and survival of most TSG101-overexpressing mammary adenosquamous carcinoma cells are dependent on the sustained expression of this oncoprotein, the activation of PI3K/AKT signaling, as well as expression of MDM2, Cyclin D1, and p21.

## Discussion

The results of this study provide experimental evidence that, when persistently overexpressed, TSG101 promotes the initiation of metaplastic lesions in the mammary epithelium and the development of triple-negative adenosquamous carcinomas. Moreover, excess levels of TSG101 are required for the survival of carcinoma cells in vivo. This suggests that TSG101 has persistent pro-tumorigenic functions during cancer initiation, progression, and maintenance of established tumors. An interesting phenomenon is the predominant occurrence of ductal hyperplasia, AMEs, and palpable adenosquamous tumors in the specific region of the mammary glands of parous mice (i.e., epithelial cells of the main collecting ducts), where the overexpression of TSG101 in prepubescent females initially led to a delay in the normal development of the gland. This observation and the higher expression of exogenous TSG101 in tumors compared to the normal mammary glands of transgenic mice might indicate that the initiation of ductal precursor lesions and carcinomas relies on an alteration in the posttranslational autoregulatory feedback loop that normally restricts the level of the TSG101 protein [30]. Although the precise mechanism controlling this feedback loop remains to be elucidated, this process is likely associated with one or several epigenetic changes that are augmented during the gestation cycle, such as DNA and histone methylation, as reviewed by Holliday et al. [56]. This would explain why the reproductive status of a female is the primary determinant for the TSG101-induced formation of large mammary tumors.

While the hyperplastic lesions within extended mammary ducts of TSG101-overexpressing mice were similar to AMEs, the palpable mammary tumors presented as adenosquamous carcinomas, sometimes retaining features of AMEs in focal areas. These findings support the previously stated notion by Foschini et al. [57] that both types of neoplasms may follow a common developmental trajectory. Nonetheless, the pathogenesis of AMEs and adenosquamous carcinomas remains poorly understood. The presence of luminal and basal epithelial cells within AMEs was regarded as evidence that these lesions may co-evolve from both epithelial cell lineages [58]. In contrast, the preservation of the cellular heterogeneity in rare cases of metastatic tumors arising from AMEs may suggest that they originate from a dual-lineage progenitor [59]. Using an H2B-GFP reporter to monitor MMTV-tTA-mediated activation of TetO-driven responder transgenes on the cellular level, we determined that the overexpression of TSG101 promotes a basal trans-differentiation of a subset of luminal epithelial cells within the ductal epithelium. Like transgenic mice that express the Flp recombinase under the control of the same MMTV promoter [38], the activation of the MMTV-tTA is typically restricted to the luminal epithelium. The atypical expression pattern of the MMTV-tTA-driven H2B-GFP reporter in basal epithelial cells within early TSG101-overexpressing preneoplastic lesions is retained in adenosquamous carcinomas. The developmental trajectory of TSG101-overexpressing tumors is quite similar to the basal trans-differentiation of luminal progenitors during the initial stages of basal-like and claudin-low mammary tumorigenesis [36, 60]. This might explain why a subset of low-grade adenosquamous carcinomas have the propensity to progress into high-grade triple-negative metaplastic breast cancer [61].

On the mechanistic level, the acquisition of basal-like characteristics instigated by the overexpression of TSG101 might be a consequence of the activation of Wnt signaling. Unsupervised hierarchical clustering indicated that TSG101-induced tumors were similar to tumors that develop late in MMTV-Wnt1 transgenic mice (Wnt1-Late^Ex^) and shared similar gene expression patterns within the luminal and basal gene subclusters [47]. Wnt signaling has been reported to suppress luminal epithelial cell lineage determination through various molecular mechanisms [62]. Although Wnt promotes the spontaneous onset of mammary tumors, recent work by Schachter et al. [63] demonstrated that the pro-tumorigenic function of Wnt is independent of its ability to trigger squamous differentiation. It is therefore possible that the activation of Wnt signaling in TSG101-induced adenosquamous carcinomas promotes cellular features independently of other oncogenic functions of TSG101. Interestingly, nearly 70% of Wnt1-Late^Ex^ mammary tumors carried mutations in codon 61 of the *Hras* gene [47], which, in association with other PI3K/AKT pathway alterations, have also been recently identified as a main driver in the pathogenesis of AMEs in human [59]. While we have not identified hotspot mutations in *Hras* and *Pik3ca*, TSG101-overexpressing tumors have elevated levels of receptor tyrosine kinases (e.g., EGFR, ERBB2/3), HRAS, and exhibit a robust activation of PI3K/AKT signaling. More importantly, the activation of the PI3K/AKT pathway and the survival of cancer cells was dependent on the sustained overexpression of TSG101 in tumors. Similar to the ablation of exogenous TSG101 in mammary tumors, the conditional knockout of TSG101 led to the downregulation of the steady-state protein expression of ERBB receptors prior to cell death [17]. As part of its function in intra-cellular trafficking, TSG101 mediates the recycling of receptor tyrosine kinases, in part, by binding to the RAB11-family interacting proteins FIP3/4 [64]. This proposed function in the modulation of receptor signaling is supported by the observation that a knockout of TSG101 disrupts the association of early endosomes from RAB11-positive recycling endosomes [1]. The effects of the downregulation of exogenous TSG101 on the decelerated growth and eventual regression of the tumors is likely mediated, in part, by the observed significant reduction in the expression of Cyclin D1. The results of this study also provide in vivo evidence for additional functions of TSG101 in cell cycle control through upregulation of MDM2 [65] as well as p21^Cip^, which does not seem to exert a growth inhibitory role in mammary tumors as previously reported for HEK293F cells and differentiated keratinocytes [9].

## Conclusions

Collectively, this work provides experimental evidence that the protein encoded by the *Tumor susceptibility gene 101* has potent oncogenic functions in vivo. The overexpression of TSG101 causes adenosquamous carcinomas that originate from luminal epithelial cells of the main mammary ducts after a median latency of 10 months. It is interesting to note that, initially, the upregulated expression of TSG101 is growth inhibitory to developing mammary ducts. Therefore, the ability of TSG101 to promote malignant transformation and cellular heterogeneity within the mammary ductal epithelium likely requires secondary events (e.g., epigenetic changes) that diminish the posttranslational autoregulatory control of the TSG101 protein expression and that enable the survival of cells expressing elevated levels of TSG101. This might explain why the expression of the TSG101 protein is significantly higher in many human breast cancer cell lines compared to untransformed epithelial cells despite similar mRNA expression levels. Consistent with findings from previous reports that TSG101 is essential for the growth of breast cancer cells in culture [25, 26], the results from this study demonstrate a perpetual pro-tumorigenic function of TSG101 for the proliferation and survival of adenosquamous carcinoma cells in established tumors, suggesting that TSG101 might serve as a rational molecular target to prevent and treat a subset of mammary tumors.

## Supplementary Information

Supplementary material 1.

## Data Availability

The RNA Sequencing data was deposited in the Gene Expression Omnibus (GEO) under accession number GSE271036. Software used for RNA sequencing analyses is described above and is freely available through Bioconductor, CRAN, or GitHub. Source data are provided in this paper. All the other data supporting the findings of this study are available from the corresponding author upon reasonable request.

## Abbreviations

AME: Adenomyoepithelioma
AKT: Serine/threonine-specific protein kinase B
Cre: Cre recombinase from the P1 bacteriophage
CK: Cytokeratin
Dox: Doxycycline
EGFR: Epidermal growth factor receptor
ERBB: Erythroblastic leukemia viral oncogene, receptor tyrosine kinase family
Flp: Flippase, site-specific recombinase from *Saccharomyces cerevisiae*
GFP: Green fluorescent protein
H2B: Histone H2B
HRAS: Harvey rat sarcoma virus transforming protein
IRES: Internal ribosome entry site
JAK: Janus tyrosine kinase
Neu: Receptor tyrosine-protein kinase ERBB2, HER2
STAT: Signal Transducer and Activator of Transcription
TetO: Tetracycline-controlled operator/promoter
Trp53: Transformation-related protein 53, TP53, p53
TSG101: Tumor susceptibility gene 101
MDM2: Mouse double minute 2 homolog
MMTV: Mouse mammary tumor virus
PI3K: Phosphoinositide 3-kinase
PyMT: Polyoma virus middle T antigen
tTA: Tetracycline-controlled transactivator
VPS: Vacuolar protein sorting
WAP: Whey acidic protein, milk protein
Wnt: Wingless, integration site 1 (int1), signal transduction pathway

## References

[1] Ferraiuolo RM, Manthey KC, Stanton MJ, Triplett AA, Wagner KU. The multifaceted roles of the tumor susceptibility gene 101 (TSG101) in normal development and disease. Cancers. 2020;12(2):450. 32075127 10.3390/cancers12020450 PMC7073217

[2] Li Y, Kane T, Tipper C, Spatrick P, Jenness DD. Yeast mutants affecting possible quality control of plasma membrane proteins. MolCell Biol. 1999;19(5):3588–99. 10.1128/mcb.19.5.3588 PMC84152 10207082

[3] Babst M, Odorizzi G, Estepa EJ, Emr SD. Mammalian tumor susceptibility gene 101 (TSG101) and the yeast homologue, Vps23p, both function in late endosomal trafficking. Traffic. 2000;1(3):248–58. 11208108 10.1034/j.1600-0854.2000.010307.x

[4] Katzmann DJ, Babst M, Emr SD. Ubiquitin-dependent sorting into the multivesicular body pathway requires the function of a conserved endosomal protein sorting complex ESCRT-I. Cell. 2001;106(2):145–55. 11511343 10.1016/s0092-8674(01)00434-2

[5] Thery C, Boussac M, Veron P, Ricciardi-Castagnoli P, Raposo G, Garin J, Amigorena S. Proteomic analysis of dendritic cell-derived exosomes: a secreted subcellular compartment distinct from apoptotic vesicles. J Immunol. 2001;166(12):7309–18. 11390481 10.4049/jimmunol.166.12.7309

[6] Colombo M, Moita C, van Niel G, Kowal J, Vigneron J, Benaroch P, Manel N, Moita LF, Thery C, Raposo G. Analysis of ESCRT functions in exosome biogenesis, composition and secretion highlights the heterogeneity of extracellular vesicles. J Cell Sci. 2013;126(Pt 24):5553–65. 24105262 10.1242/jcs.128868

[7] Hittelman AB, Burakov D, Iniguez-Lluhi JA, Freedman LP, Garabedian MJ. Differential regulation of glucocorticoid receptor transcriptional activation via AF-1-associated proteins. EMBO J. 1999;18(19):5380–8. 10508170 10.1093/emboj/18.19.5380 PMC1171607

[8] Lin YS, Chen YJ, Cohen SN, Cheng TH. Identification of TSG101 functional domains and p21 loci required for TSG101-mediated p21 gene regulation. PLoS ONE. 2013;8(11): e79674. 24244542 10.1371/journal.pone.0079674 PMC3823576

[9] Oh H, Mammucari C, Nenci A, Cabodi S, Cohen SN, Dotto GP. Negative regulation of cell growth and differentiation by TSG101 through association with p21Cip1/WAF1. ProcNatlAcadSciUSA. 2002;99(8):5430–5. 10.1073/pnas.082123999 PMC122786 11943869

[10] Zhong Q, Chen Y, Jones D, Lee WH. Perturbation of TSG101 protein affects cell cycle progression. Cancer Res. 1998;58:2699–702. 9661875

[11] Lee HH, Elia N, Ghirlando R, Lippincott-Schwartz J, Hurley JH. Midbody targeting of the ESCRT machinery by a noncanonical coiled coil in CEP55. Science. 2008;322(5901):576–80. 18948538 10.1126/science.1162042 PMC2720046

[12] Wagner KU, Dierisseau P, Rucker EB, Robinson GW, Hennighausen L. Genomic architecture and transcriptional activation of the mouse and human tumor susceptibility gene TSG101: common types of shorter transcripts are true alternative splice variants. Oncogene. 1998;17:2761–70. 9840940 10.1038/sj.onc.1202529

[13] Ruland J, Sirard C, Elia A, MacPherson D, Wakeham A, Li L, Luis DLP, Cohen SN, Mak TW. p53 Accumulation, defective cell proliferation, and early embryonic lethality in mice lacking tsg101. ProcNatlAcadSciUSA. 2001;98(4):1859–64. 10.1073/pnas.98.4.1859 PMC29347 11172041

[14] Wagner KU, Krempler A, Qi Y, Park K, Henry MD, Triplett AA, Riedlinger G, Rucker EB III, Hennighausen L. Tsg101 Is essential for cell growth, proliferation, and cell survival of embryonic and adult tissues. MolCell Biol. 2003;23(1):150–62. 10.1128/MCB.23.1.150-162.2003 PMC140677 12482969

[15] Carstens MJ, Krempler A, Triplett AA, van Lohuizen M, Wagner KU. Cell cycle arrest and cell death are controlled by p53-dependent and p53-independent mechanisms in Tsg101-deficient cells. JBiolChem. 2004;279(34):35984–94. 10.1074/jbc.M400408200 PMC1201394 15210712

[16] Krempler A, Henry MD, Triplett AA, Wagner KU. Targeted deletion of the Tsg101 gene results in cell cycle arrest at G1/S and p53-independent cell death. JBiolChem. 2002;277(45):43216–23. 10.1074/jbc.M207662200 PMC1201509 12205095

[17] Morris CR, Stanton MJ, Manthey KC, Oh KB, Wagner KU. A knockout of the Tsg101 gene leads to decreased expression of ErbB receptor tyrosine kinases and induction of autophagy prior to cell death. PLoSOne. 2012;7(3): e34308. 10.1371/journal.pone.0034308 PMC3316634 22479596

[18] Li L, Cohen SN. Tsg101: a novel tumor susceptibility gene isolated by controlled homozygous functional knockout of allelic loci in mammalian cells. Cell. 1996;85:319–29. 8616888 10.1016/s0092-8674(00)81111-3

[19] Liu RT, Huang CC, You HL, Chou FF, Hu CC, Chao FP, Chen CM, Cheng JT. Overexpression of tumor susceptibility gene TSG101 in human papillary thyroid carcinomas. Oncogene. 2002;21(31):4830–7. 12101421 10.1038/sj.onc.1205612

[20] Oh KB, Stanton MJ, West WW, Todd GL, Wagner KU. Tsg101 is upregulated in a subset of invasive human breast cancers and its targeted overexpression in transgenic mice reveals weak oncogenic properties for mammary cancer initiation. Oncogene. 2007;26(40):5950–9. 17369844 10.1038/sj.onc.1210401

[21] Liu F, Yu Y, Jin Y, Fu S. TSG101, identified by screening a cancer cDNA library and soft agar assay, promotes cell proliferation in human lung cancer. MolBiolRep. 2010;37(6):2829–38. 10.1007/s11033-009-9835-5 19787439

[22] Young TW, Rosen DG, Mei FC, Li N, Liu J, Wang XF, Cheng X. Up-regulation of tumor susceptibility gene 101 conveys poor prognosis through suppression of p21 expression in ovarian cancer. ClinCancer Res. 2007;13(13):3848–54. 10.1158/1078-0432.CCR-07-0337 17606716

[23] Young TW, Mei FC, Rosen DG, Yang G, Li N, Liu J, Cheng X. Up-regulation of tumor susceptibility gene 101 protein in ovarian carcinomas revealed by proteomics analyses. Mol Cell Proteomics. 2007;6(2):294–304. 17110434 10.1074/mcp.M600305-MCP200

[24] Ma XR, Edmund Sim UH, Pauline B, Patricia L, Rahman J. Overexpression of WNT2 and TSG101 genes in colorectal carcinoma. TropBiomed. 2008;25(1):46–57. 18600204

[25] Zhu G, Gilchrist R, Borley N, Chng HW, Morgan M, Marshall JF, Camplejohn RS, Muir GH, Hart IR. Reduction of TSG101 protein has a negative impact on tumor cell growth. IntJCancer. 2004;109(4):541–7. 10.1002/ijc.20014 14991575

[26] Zhang Y, Song M, Cui ZS, Li CY, Xue XX, Yu M, Lu Y, Zhang SY, Wang EH, Wen YY. Down-regulation of TSG101 by small interfering RNA inhibits the proliferation of breast cancer cells through the MAPK/ERK signal pathway. Histol Histopathol. 2011;26(1):87–94. 21117030 10.14670/HH-26.87

[27] Xu C, Zheng J. siRNA against TSG101 reduces proliferation and induces G0/G1 arrest in renal cell carcinoma - involvement of c-myc, cyclin E1, and CDK2. Cell Mol Biol Lett. 2019;24:7. 30675171 10.1186/s11658-018-0124-y PMC6332891

[28] Shao Z, Ji W, Liu A, Qin A, Shen L, Li G, Zhou Y, Hu X, Yu E, Jin G. TSG101 silencing suppresses hepatocellular carcinoma cell growth by inducing cell cycle arrest and autophagic cell death. Med Sci Monit. 2015;21:3371–9. 26537625 10.12659/MSM.894447 PMC4654595

[29] Henry MD, Triplett AA, Oh KB, Smith GH, Wagner KU. Parity-induced mammary epithelial cells facilitate tumorigenesis in MMTV-neu transgenic mice. Oncogene. 2004;23(41):6980–5. 15286714 10.1038/sj.onc.1207827

[30] Feng GH, Lih CJ, Cohen SN. TSG101 protein steady-state level is regulated posttranslationally by an evolutionarily conserved COOH-terminal sequence. Cancer Res. 2000;60(6):1736–41. 10749147

[31] Essandoh K, Deng S, Wang X, Jiang M, Mu X, Peng J, Li Y, Peng T, Wagner KU, Rubinstein J, et al. Tsg101 positively regulates physiologic-like cardiac hypertrophy through FIP3-mediated endosomal recycling of IGF-1R. FASEB J of American Societies Experiment Biol. 2019;33(6):7451–66. 10.1096/fj.201802338RR PMC6529337 30884248

[32] Oh KBSM, West WW, Todd GL, Wagner KU. Tsg101 is upregulated in a subset of invasive breast cancers and its targeted overexpression in transgenic mice reveals weak oncogenic properties for mammary cancer initiation. Oncogene. 2007;26:5950–9. 17369844 10.1038/sj.onc.1210401

[33] Lin WC, Schmidt JW, Creamer BA, Triplett AA, Wagner KU. Gain-of-function of Stat5 leads to excessive granulopoiesis and lethal extravasation of granulocytes to the lung. PLoSOne. 2013;8(4): e60902. 10.1371/journal.pone.0060902 PMC3614894 23565285

[34] Sakamoto K, Schmidt JW, Wagner KU. Generation of a novel MMTV-tTA transgenic mouse strain for the targeted expression of genes in the embryonic and postnatal mammary gland. PLoSOne. 2012;7(8): e43778. 10.1371/journal.pone.0043778 PMC3432039 22952764

[35] Zhang Q, Sakamoto K, Wagner KU. D-type Cyclins are important downstream effectors of cytokine signaling that regulate the proliferation of normal and neoplastic mammary epithelial cells. Mol Cell Endocrinol. 2014;382(1):583–92. 23562856 10.1016/j.mce.2013.03.016 PMC3740091

[36] Rädler PD, Wehde BL, Triplett AA, Shrestha H, Shepherd JH, Pfefferle AD, Rui H, Cardiff RD, Perou CM, Wagner KU. Highly metastatic claudin-low mammary cancers can originate from luminal epithelial cells. Nat Commun. 2021;12(1):3742. 34145248 10.1038/s41467-021-23957-5 PMC8213728

[37] Tumbar T, Guasch G, Greco V, Blanpain C, Lowry WE, Rendl M, Fuchs E. Defining the epithelial stem cell niche in skin. Science. 2004;303(5656):359–63. 14671312 10.1126/science.1092436 PMC2405920

[38] Rädler PD, Vistisen K, Triplett AA, Dennaoui R, Li Y, Shrestha H, Ferraiuolo R-M, Thangasamy A, Saur D, Wagner K-U. Dual recombinase action in the normal and neoplastic mammary gland epithelium. Sci Rep. 2021;11(1):20775. 34675248 10.1038/s41598-021-00231-8 PMC8531329

[39] Sousa VH, Miyoshi G, Hjerling-Leffler J, Karayannis T, Fishell G. Characterization of Nkx6-2-derived neocortical interneuron lineages. Cereb Cortex. 2009;19(Suppl 1):i1-10. 19363146 10.1093/cercor/bhp038 PMC2693535

[40] Sakamoto K, Lin WC, Triplett AA, Wagner KU. Targeting janus kinase 2 in Her2/neu-expressing mammary cancer: implications for cancer prevention and therapy. Cancer Res. 2009;69(16):6642–50. 19638583 10.1158/0008-5472.CAN-09-0746 PMC2758773

[41] Zhang Q, Sakamoto K, Liu C, Triplett AA, Lin WC, Rui H, Wagner KU. Cyclin D3 compensates for the loss of cyclin D1 during ErbB2-induced mammary tumor initiation and progression. Cancer Res. 2011;71(24):7513–24. 22037875 10.1158/0008-5472.CAN-11-1783 PMC3242818

[42] Liao Y, Smyth GK, Shi W. The R package Rsubread is easier, faster, cheaper and better for alignment and quantification of RNA sequencing reads. Nucleic Acids Res. 2019;47(8): e47. 30783653 10.1093/nar/gkz114 PMC6486549

[43] Robinson MD, McCarthy DJ, Smyth GK. edgeR: a Bioconductor package for differential expression analysis of digital gene expression data. Bioinformatics (Oxford, England). 2010;26(1):139–40. 19910308 10.1093/bioinformatics/btp616 PMC2796818

[44] Yu G, Wang LG, Han Y, He QY. clusterProfiler: an R package for comparing biological themes among gene clusters. OMICS. 2012;16(5):284–7. 22455463 10.1089/omi.2011.0118 PMC3339379

[45] Hollern DP, Xu N, Thennavan A, Glodowski C, Garcia-Recio S, Mott KR, He X, Garay JP, Carey-Ewend K, Marron D, et al. B cells and T follicular helper cells mediate response to checkpoint inhibitors in high mutation burden mouse models of breast cancer. Cell. 2019;179(5):1191-1206.e1121. 31730857 10.1016/j.cell.2019.10.028 PMC6911685

[46] Lim C, Hwang D, Yazdimamaghani M, Atkins HM, Hyun H, Shin Y, Ramsey JD, Rädler PD, Mott KR, Perou CM, et al. High-Dose paclitaxel and its combination with CSF1R inhibitor in polymeric micelles for chemoimmunotherapy of triple negative breast cancer. Nano Today. 2023;51:101884. 37484164 10.1016/j.nantod.2023.101884 PMC10357922

[47] Pfefferle AD, Darr DB, Calhoun BC, Mott KR, Rosen JM, Perou CM. The MMTV-Wnt1 murine model produces two phenotypically distinct subtypes of mammary tumors with unique therapeutic responses to an EGFR inhibitor. Disease Models Mech. 2019. 10.1242/dmm.037192. 10.1242/dmm.037192 PMC6679375 31213486

[48] de Hoon MJ, Imoto S, Nolan J, Miyano S. Open source clustering software. Bioinformatics (Oxford, England). 2004;20(9):1453–4. 14871861 10.1093/bioinformatics/bth078

[49] Saldanha AJ. Java Treeview–extensible visualization of microarray data. Bioinformatics (Oxford, England). 2004;20(17):3246–8. 15180930 10.1093/bioinformatics/bth349

[50] Doyotte A, Russell MR, Hopkins CR, Woodman PG. Depletion of TSG101 forms a mammalian “Class E” compartment: a multicisternal early endosome with multiple sorting defects. JCell Sci. 2005;118(Pt 14):3003–17. 16014378 10.1242/jcs.02421

[51] Bache KG, Slagsvold T, Cabezas A, Rosendal KR, Raiborg C, Stenmark H. The growth-regulatory protein HCRP1/hVps37A is a subunit of mammalian ESCRT-I and mediates receptor down-regulation. Mol Biol Cell. 2004;15(9):4337–46. 15240819 10.1091/mbc.E04-03-0250 PMC515363

[52] Adams JR, Xu K, Liu JC, Agamez NM, Loch AJ, Wong RG, Wang W, Wright KL, Lane TF, Zacksenhaus E, et al. Cooperation between Pik3ca and p53 mutations in mouse mammary tumor formation. Can Res. 2011;71(7):2706–17. 10.1158/0008-5472.CAN-10-0738 21324922

[53] Meyer DS, Brinkhaus H, Muller U, Muller M, Cardiff RD, Bentires-Alj M. Luminal expression of PIK3CA mutant H1047R in the mammary gland induces heterogeneous tumors. Cancer Res. 2011;71(13):4344–51. 21482677 10.1158/0008-5472.CAN-10-3827

[54] Gross JC, Chaudhary V, Bartscherer K, Boutros M. Active Wnt proteins are secreted on exosomes. Nat Cell Biol. 2012;14(10):1036–45. 22983114 10.1038/ncb2574

[55] Jung HH, Kim J-Y, Lim JE, Im Y-H. Cytokine profiling in serum-derived exosomes isolated by different methods. Sci Rep. 2020;10(1):14069. 32826923 10.1038/s41598-020-70584-z PMC7442638

[56] Holliday H, Baker LA, Junankar SR, Clark SJ, Swarbrick A. Epigenomics of mammary gland development. Breast cancer research : BCR. 2018;20(1):100. 30176939 10.1186/s13058-018-1031-x PMC6122685

[57] Foschini MP, Pizzicannella G, Peterse JL, Eusebi V. Adenomyoepithelioma of the breast associated with low-grade adenosquamous and sarcomatoid carcinomas. Virchows Archiv: Int J Pathol. 1995;427(3):243–50. 10.1007/BF00203390 7496592

[58] Rakha E, Tan PH, Ellis I, Quinn C. Adenomyoepithelioma of the breast: a proposal for classification. Histopathology. 2021;79(4):465–79. 33829532 10.1111/his.14380

[59] Geyer FC, Li A, Papanastasiou AD, Smith A, Selenica P, Burke KA, Edelweiss M, Wen H-C, Piscuoglio S, Schultheis AM, et al. Recurrent hotspot mutations in HRAS Q61 and PI3K-AKT pathway genes as drivers of breast adenomyoepitheliomas. Nat Commun. 2018;9(1):1816. 29739933 10.1038/s41467-018-04128-5 PMC5940840

[60] Lim E, Vaillant F, Wu D, Forrest NC, Pal B, Hart AH, Asselin-Labat ML, Gyorki DE, Ward T, Partanen A, et al. Aberrant luminal progenitors as the candidate target population for basal tumor development in BRCA1 mutation carriers. NatMed. 2009;15(8):907–13. 10.1038/nm.2000 19648928

[61] Kawachi K, Tang X, Kasajima R, Yamanaka T, Shimizu E, Katayama K, Yamaguchi R, Yokoyama K, Yamaguchi K, Furukawa Y, et al. Genetic analysis of low-grade adenosquamous carcinoma of the breast progressing to high-grade metaplastic carcinoma. Breast Cancer Res Treat. 2023;202(3):563–73. 37650999 10.1007/s10549-023-07078-9 PMC10564816

[62] Wicker MN, Wagner KU. Cellular plasticity in mammary gland development and breast cancer. Cancers. 2023;15(23):5605. 38067308 10.3390/cancers15235605 PMC10705338

[63] Schachter NF, Adams JR, Skowron P, Kozma KJ, Lee CA, Raghuram N, Yang J, Loch AJ, Wang W, Kucharczuk A, et al. Single allele loss-of-function mutations select and sculpt conditional cooperative networks in breast cancer. Nat Commun. 2021;12(1):5238. 34475389 10.1038/s41467-021-25467-w PMC8413298

[64] Horgan CP, Hanscom SR, Kelly EE, McCaffrey MW. Tumor susceptibility gene 101 (TSG101) is a novel binding-partner for the class II Rab11-FIPs. PLoS ONE. 2012;7(2): e32030. 22348143 10.1371/journal.pone.0032030 PMC3279423

[65] Li L, Liao J, Ruland J, Mak TW, Cohen SN. A TSG101/MDM2 regulatory loop modulates MDM2 degradation and MDM2/p53 feedback control. ProcNatlAcadSciUSA. 2001;98(4):1619–24. 10.1073/pnas.98.4.1619 PMC29306 11172000

