# Supplementary material for: Overexpression of TSG101 causes the development of adenosquamous mammary carcinoma
Source: Breast Cancer Res. 2025 Jul 7;27:126. doi: 10.1186/s13058-025-02007-8 (PMC12232759; doi:10.1186/s13058-025-02007-8)

Supplemental information

Overexpression of TSG101 causes the development of adenosquamous mammary carcinoma

Rayane Dennaoui, Patrick D. Rädler, Madison Wicker, Kerry Vistisen, Rosa-Maria Ferraiuolo, Aleata A. Triplett, Hridaya Shrestha, Tessa A. Liner; Karoline C. Manthey, Hallgeir Rui, Robert D. Cardiff, Teresa M. Gunn, Charles M. Perou, and Kay-Uwe Wagner

Supplemental Figures S1-S8

Supplementary Tables 1 and 2

Uncropped images of immunoblots

Supplemental Fig. S1 Dennaoui et al.

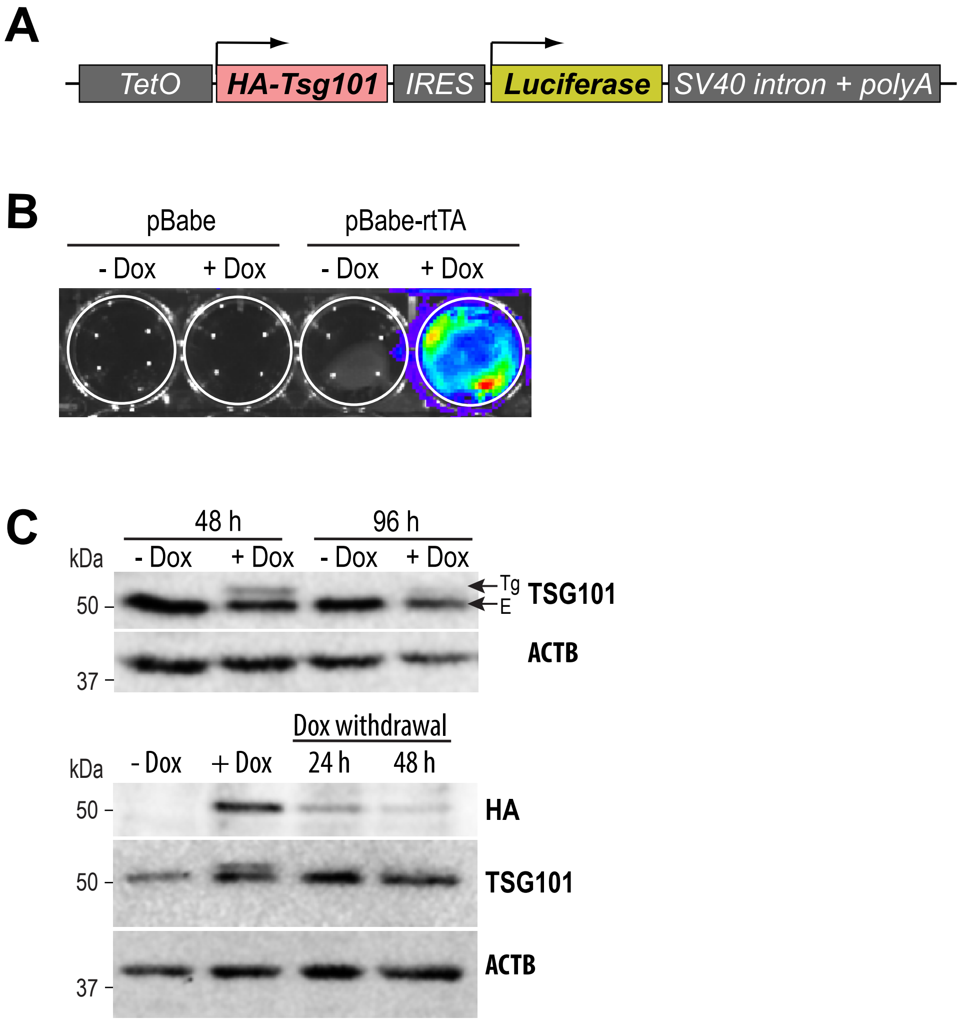

Supplemental Figure S1. Generation of transgenic mice expressing TSG101 in a ligand-controlled manner

A. Schematic outline of the TetO-Tsg101 transgene. The full-length mouse TSG101 with an N-terminal hemagglutinin (HA) tag is expressed along with the luciferase reporter upon binding of the tetracycline-controlled transactivator (tTA or rtTA) to its operator/promoter (TetO); IRES, internal ribosomal entry site; SV40 poly A, intron and polyadenylation signal from the Simian-Virus 40.

B. Bioluminescence imaging of explanted mouse embryonic fibroblasts from TetO-Tsg101 transgenic line 1 that were infected with a pBabe-puro retrovirus expressing the reverse tetracycline-controlled transactivator (rtTA) and treated with doxycycline (Dox, a tetracycline derivative). The rtTA-mediated expression of luciferase in the presence of Dox (tet-on system) confirms the stringent control of the ligand-mediated expression of the transgene.

C. Western blot analysis to validate the Dox-dependent expression of exogenous, HA-tagged TSG101 from the transgene and its downregulation following the withdrawal of the ligand. Beta-Actin (ACTB) served as a loading control.

Supplemental Fig. S2 Dennaoui et al.

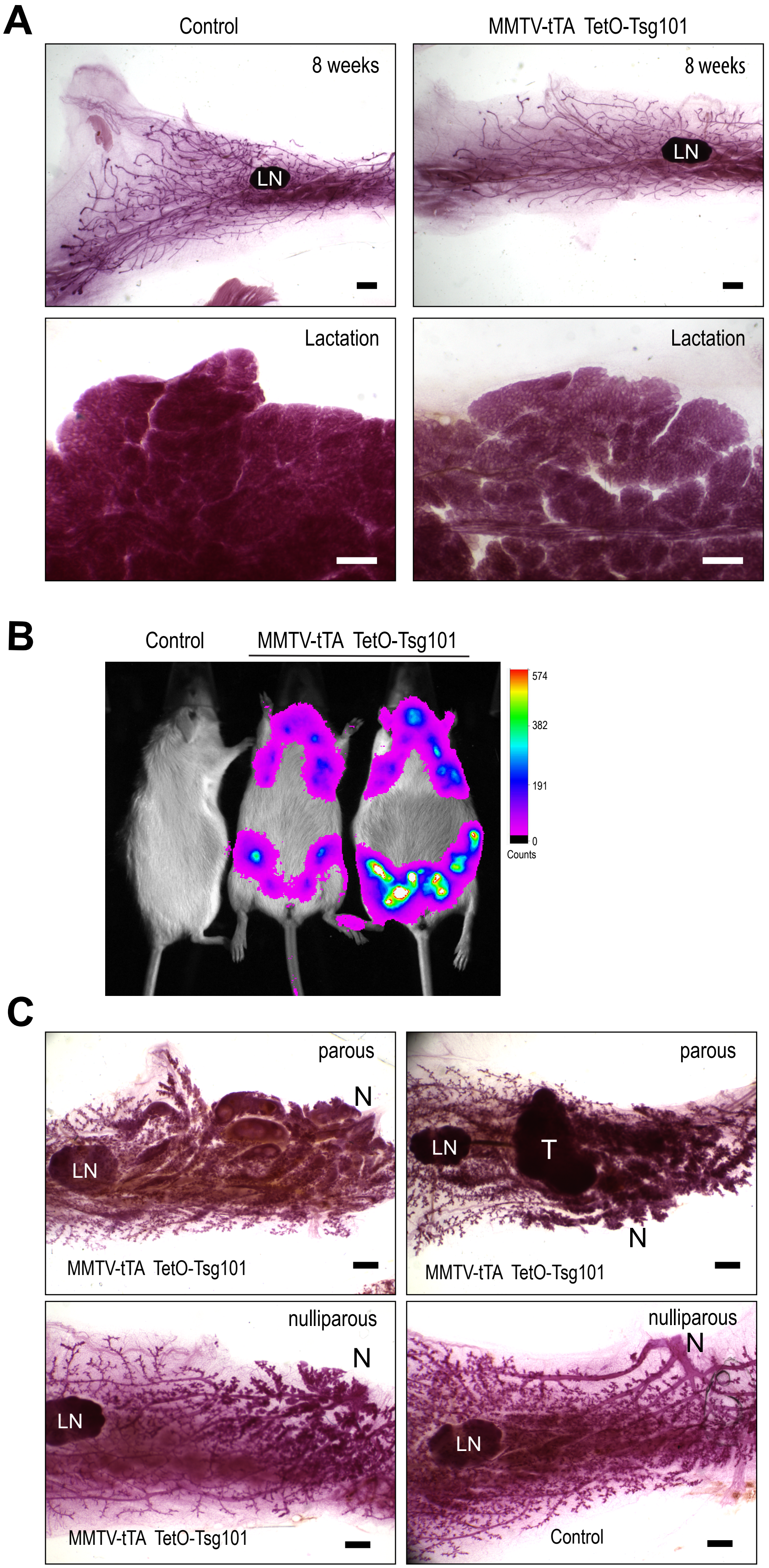

Supplemental Figure S2. The MMTV-tTA-mediated, constitutive overexpression of TSG101 does not impair the functional differentiation of the mammary gland and lactation but frequently leads to severe cases of ductal hyperplasia near the nipple region in nonpregnant, postpartum females

A. Carmine alum-stained wholemounts of mammary glands from 8-week-old nulliparous (upper panels) and 10 days lactating (lower panels) MMTV-tTA TetO-TSG101 double transgenic females and TetO-Tsg101 single transgenic controls; bars, 1 mm.

B. In vivo bioluminescence imaging (Bruker In Vivo Xtreme) of 10-day lactating double transgenic females and a control.

C. Upper panel: Carmine alum-stained wholemounts of mammary glands with severe cases of ductal hyperplasia in nonpregnant multiparous MMTV-tTA TetO-TSG101 females. Lower panel: Small hyperplastic ducts in age-matched nulliparous double transgenic females in comparison to a nulliparous wildtype control; bars, 1 mm.

Note that the MMTV-driven transactivator (tTA) induces a constitutive expression of TSG101 and luciferase without the administration of doxycycline (tet-off repressor system) during all stages of mammary gland development; LN, lymph node; N, nipple region.

Supplemental Fig. S3

A

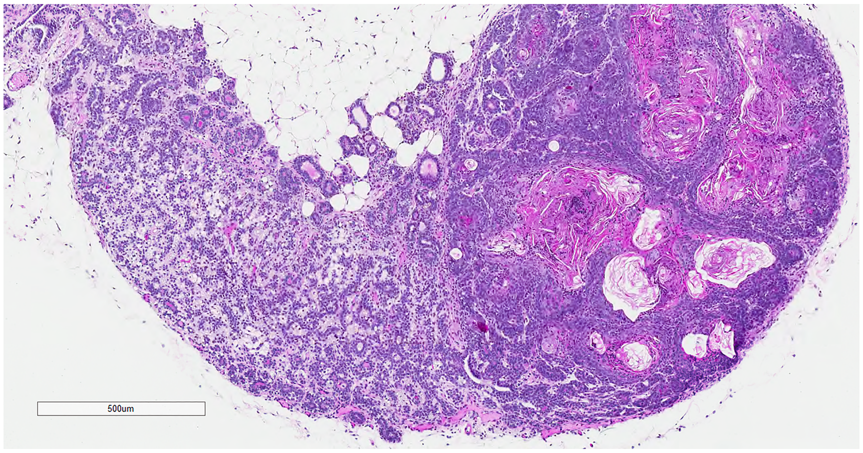

B

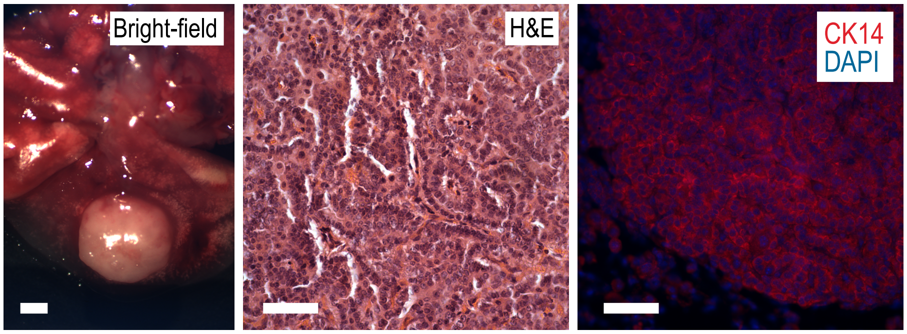

C

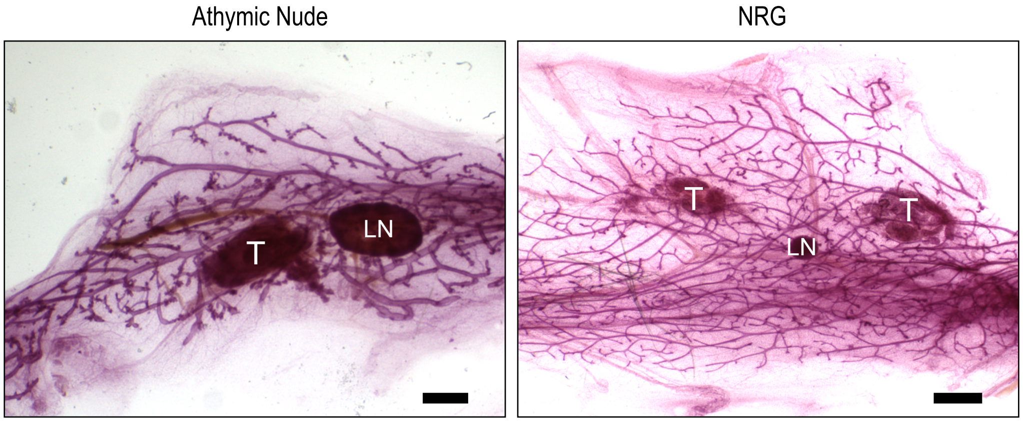

Supplemental Figure S3. TSG101-induced malignant progression of adenomyoepithelioma and adenosquamous carcinoma

A. Hematoxylin and eosin (H&E)-stained histologic section of a hyperplastic duct with a developing mammary tumor from an MMTV-tTA TetO-Tsg101 double transgenic female; bar, 500 µm.

B. Left: Stereoscopic bright-field image of a lung metastasis in a TSG101 overexpressing female; bar, 1 mm. Middle, right: H&E-stained histologic section and immunofluorescent staining of basal cytokeratin 14 (CK14) of a lung metastatic lesion; bars, 50 µm.

C. Carmine alum-stained mammary gland wholemounts of immunocompromised recipient females (athymic nude and NOD-*Rag1*^{null} *IL2rg*^{null}, NRG) that were implanted with mammary tumor fragments (T) from MMTV-tTA TetO-Tsg101 double transgenic females; bars, 1 mm; LN, lymph node.

Supplemental Fig. S4

Dennaoui et al.

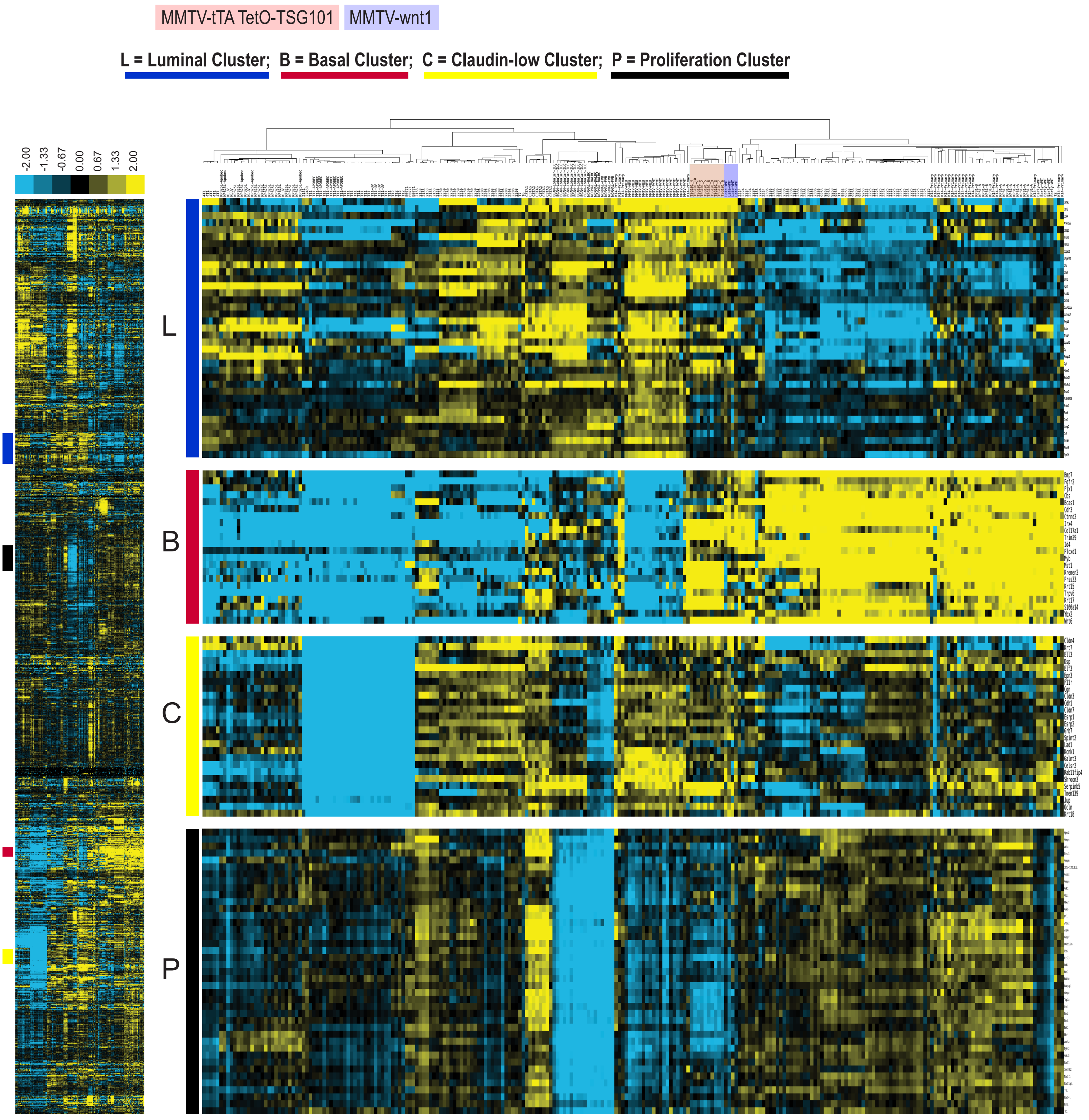

Supplemental Figure S4. Intrinsic gene cluster of TSG101 overexpressing mammary tumors in comparison to 31 reference mammary tumor models and normal mammary gland tissues Left, overview of the full gene expression cluster with 1,723 intrinsic genes from 251 individual mouse mammary tumors representing 31 reference mammary tumor models alongside normal mammary gland tissues from the FVB/N and BALB/c females. Right, gene expression subclusters with dendrogram and model names illustrating that TSG101-overexpressing tumors (light red, N=10) and cancers that originated late in MMTV-Wnt1 females (light blue, Wnt1-Late^{Ex}) cluster with luminal mammary tumors from MMTV-Neu and MMTV-PyMT mice (L, dark blue), but exhibit an upregulated expression of within the basal and claudin-low subclusters (B, red; C, yellow). Proliferation subcluster (P, black) shows that TSG101-overexpressing tumors have a relatively low proliferation rate.

Supplemental Fig. S5 Dennaoui et al.

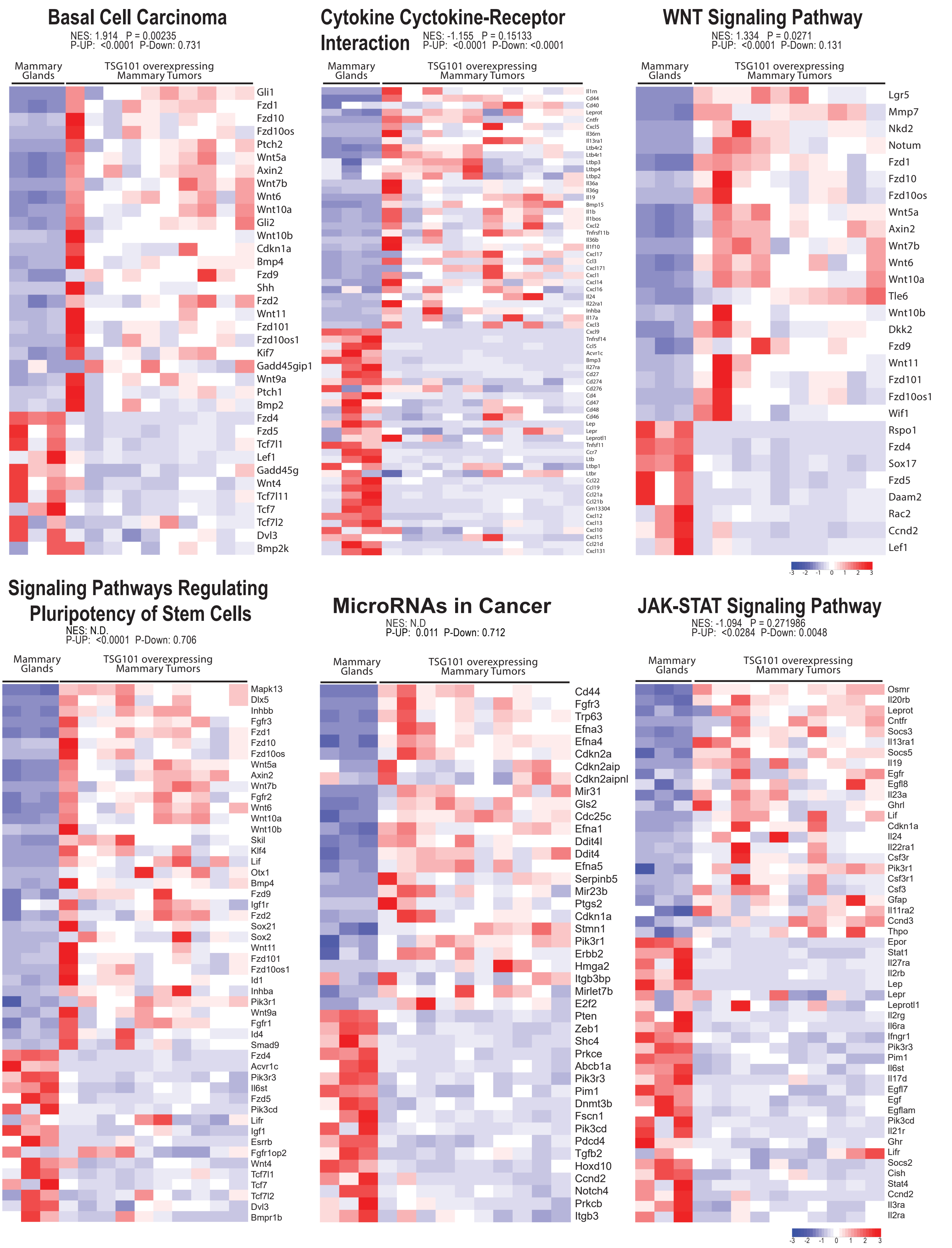

Supplemental Figure S5. Heatmaps of significantly deregulated pathways in TSG101-overexpressing mammary tumors

Molecular pathways that were significantly enriched in the gene set enrichment analysis with normal FVB mammary glands and TSG101-overexpressing tumors. Significance is given at a p-value < 0.05 for the normalized enrichment score of the entire pathway or enrichment of up/down regulated gene sets within the pathway. Genes plotted are significantly deregulated genes within those pathways.

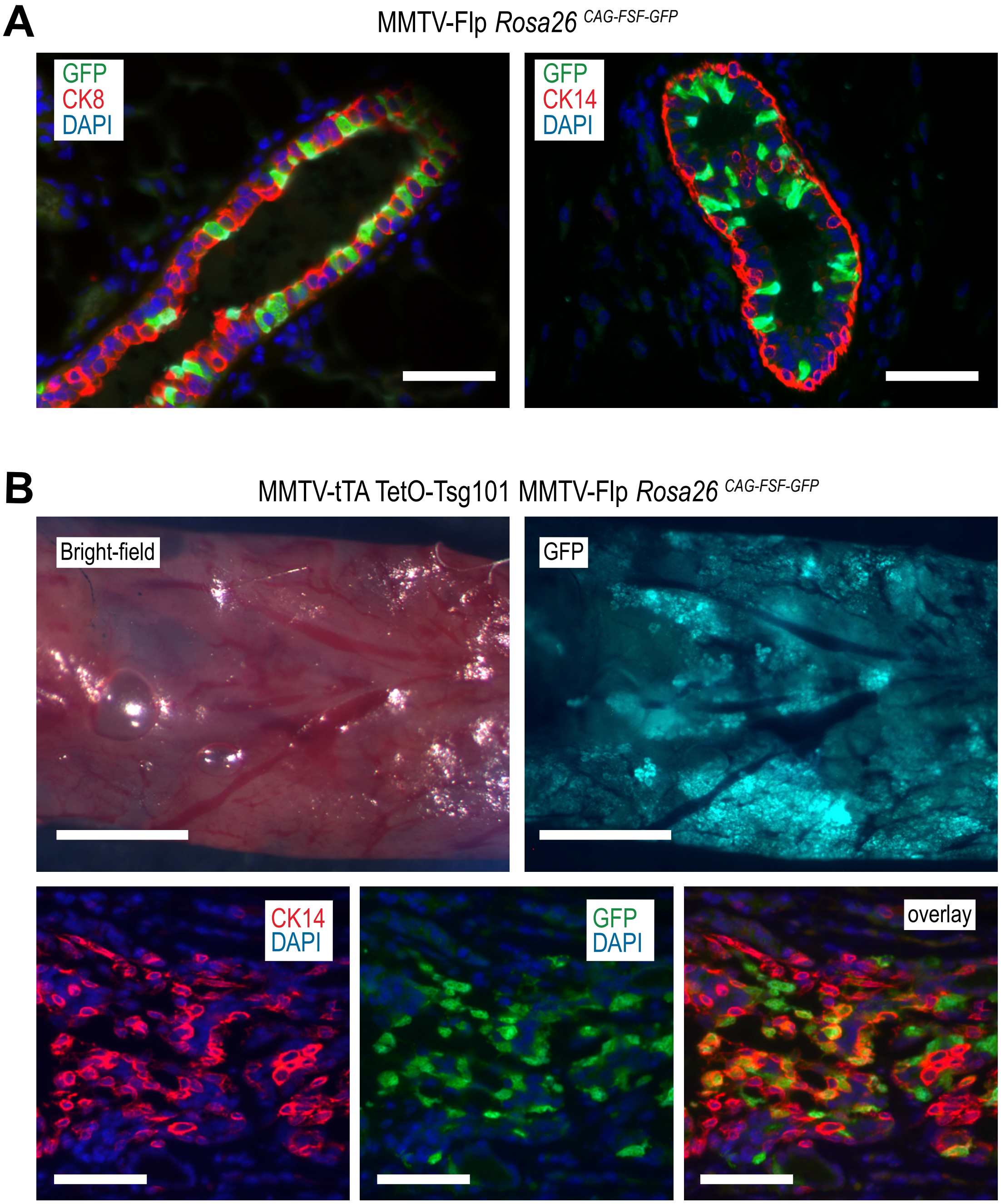
A
MMTV-Flp *Rosa26* $^{CAG\text{-}FSF\text{-}GFP}$
GFP
CK8
DAPI
GFP
CK14
DAPI
B
MMTV-tTA TetO-Tsg101 MMTV-Flp *Rosa26* $^{CAG\text{-}FSF\text{-}GFP}$
Bright-field
GFP
CK14
DAPI
GFP
DAPI
overlay

Supplemental Figure S6. Cell lineage tracing of transforming luminal epithelial cells using the MMTV-Flp in combination with the *Rosa26*$^{CAG\text{-}FSF\text{-}GFP}$ reporter

A. Immunofluorescent (IF) co-staining of cytoplasmic GFP along with luminal cytokeratin 8 (CK8) or basal cytokeratin 14 (CK14) in the mammary gland of MMTV-Flp *Rosa26*$^{CAG\text{-}FSF\text{-}GFP}$ females double transgenic females; bars 50 µm. Note that the MMTV-Flp activates the ubiquitously active *Rosa26*$^{CAG\text{-}FSF\text{-}GFP}$ reporter in a subset of luminal epithelial cells.

B. Upper panel: Stereoscopic bright-field image (left) with corresponding GFP fluorescent image (right) of a mammary gland from a parous MMTV-tTA TetO-Tsg101 MMTV-Flp *Rosa26*$^{CAG\text{-}FSF\text{-}GFP}$ female; bar, 1 mm. Lower panel: IF staining of GFP and Cytokeratin 14 (CK14) on a histologic section of a hyperplastic duct of a quadruple transgenic female; bars, 50 µm

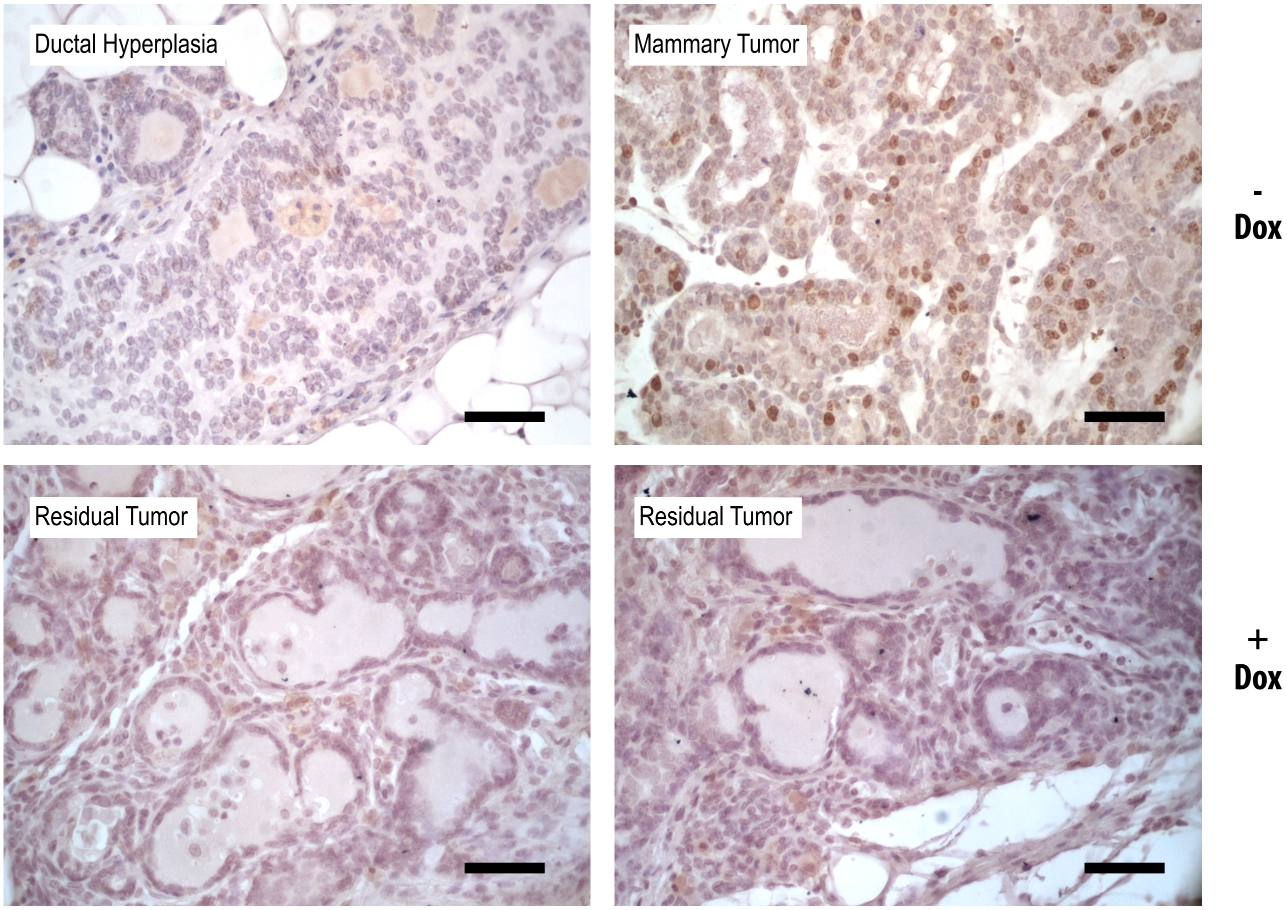
Ductal Hyperplasia
Mammary Tumor
Residual Tumor
Residual Tumor
-
Dox
+
Dox

Supplemental Figure S7. Lack of proliferation in residual tumors after the downregulation of TSG101

Immunohistochemical staining of Ki-67 on histologic sections of ductal hyperplasia and adenosquamous carcinoma (upper panel, -Dox) as well as cancer cells of residual tumor tissues from MMTV-tTA TetO-TSG101 females were treated with Dox for 30 days (+Dox). Slides were counterstained with hematoxylin, bars, 50 µm.

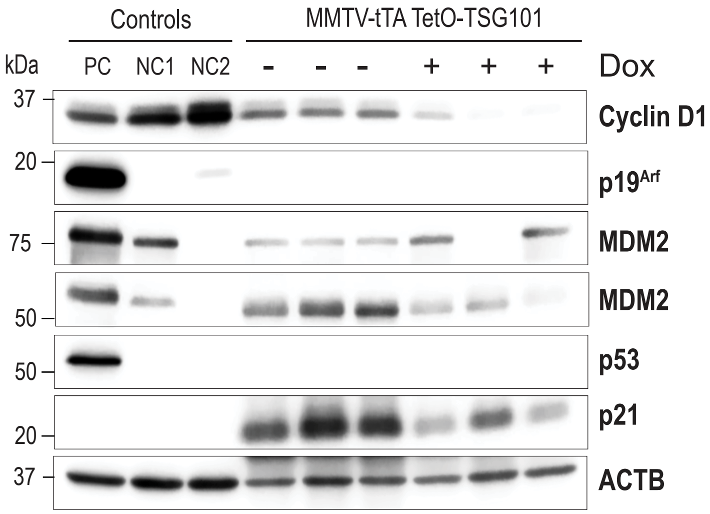

Controls

PC *MMTV-Flp KrasG12D Trp53* ^{R172H} Mammary Tumor

NC1 NIH3T3

NC2 *Mdm2* $^{-/-}$ *Trp53* $^{-/-}$ MEFs

Supplemental Figure S8. Downregulation of exogenous TSG101 in mammary tumors leads to a decrease in the steady-state expression of critical cell cycle regulators

Western blot analysis of Cyclin D1, $p19^{Arf}$, MDM2, p53, and $p21^{Cip}$ in mammary carcinomas before (-Dox) and 72 hrs of treatment with doxycycline (+Dox). Beta-actin (ACTB) was used as a loading control; PC, positive control, mutant KRAS-induced mouse mammary tumor cells expressing high levels of mutant p53, $p19^{Arf}$, and MDM2; NC, negative controls, NC1 $p19^{Arf}$ deficient NIH3T3 cells; NC2, mouse embryonic fibroblasts (MEFs) with a targeted double knockout of MDM2 and p53.

Supplementary Table 1: Primary and secondary antibodies for immunofluorescent and immunohistochemistry staining.

**Primary Antibodies**	**SOURCE**	**IDENTIFIER**	**DILUTION**
α-GFP	Avēs Labs	GFP-1020	1:500
α-CK8	Developmental Studies Hybridoma Bank	TROMAI	1:100
α-CK18	Invitrogen	61428PROGEN	1:20
α-CK14	Covance	PRB-155P	1:1000
α-CK5	Covance	PRB-160P	1:200
α-FoxA1	Invitrogen	MA5-32556	1:100
α-Ki67	Abcam	ab15580	1:200
**Secondary Antibodies**	**SOURCE**	**IDENTIFIER**	**Dilution**
Alexa Fluor 488 goat anti-chicken	Invitrogen	A11039	1:1000
Alexa Fluor 488 donkey anti-rat	Invitrogen	A21208	1:1000
Alexa Fluor 594 donkey anti-rat	Invitrogen	A21209	1:1000
Alexa Fluor 594 goat anti-rabbit	Invitrogen	A11012	1:1000
Alexa Fluor 594 donkey anti-rabbit	Invitrogen	A21207	1:200-1000
Alexa Fluor 488 donkey anti-mouse	Invitrogen	A21202	1:1000

Supplementary Table 2: Primary and secondary antibodies for immunoblotting.

Primary Antibodies	SOURCE	IDENTIFIER	DILUTION
α-TSG101	Cell Signaling	#72312	1:1000
α-TSG101	Santa Cruz	#7964	1:2000
α-HA-tag	Cell Signaling	#3724	1:1000
α-Vps28	Santa Cruz	#166537	1:1000
α-ErbB2 (HER-2)	Invitrogen	MA5-15050	1:1000
α-Estrogen Receptor alpha	Abcam	ab32063	1:1000
α-Progesterone Receptor	Invitrogen	MA1-410	1:500
α-phospho-EGF receptor	Cell Signaling	#2234	1:1000
α-EGFR	Santa Cruz	#373746	1:1000
α-ErbB3/ HER3	Santa Cruz	#7390	1:200
α-HRAS	Novus Biologicals	NBP3-15818	1:1000
α-phosphorylated ERK1/2	Cell Signaling	9101	1:1000
α- ERK1/2	BD Transduction Laboratories	#610123	1:1000
α- p110a	Cell Signaling	#4249	1:1000
α- p85 (p50/p55)	Upstate	#06-195	1:1000
α- pT308-AKT	Cell Signaling	#4056	1:1000
α- pS473-AKT	Cell Signaling	#9271	1:1000
α-AKT	Cell Signaling	#9272	1:1000
α-phospho-STAT3	Cell Signaling	#9145	1:1000
α-STAT3	Cell Signaling	#9139	1:1000
α-phospho-STAT1	Origene	TA309955	1:1000
α-STAT1	Cell Signaling	#14994	1:1000
α-phospho-STAT5	Cell Signaling	#9351	1:1000
α-STAT5	Santa Cruz	#836	1:1000
α-MDM2	Invitrogen	#700555	1:1000
α- p53	Novus Biologicals	NBP2-62555	1:1000
α- p21	BD Biosciences	#556431	1:1000
α- p19ARF	Abcam	ab80	1:1000
α- phospho-FoxO3a	Cell Signaling	#9465	1:1000
α- FoxO3a	Cell Signaling	#9467	1:1000
α- IGF-1R	Cell Signaling	#3918	1:1000
α-Cyclin D1	Abcam	ab16663	1:1000
α-GAPDH	Cell Signaling	#5174S	1:2000
α-ACTB	Santa Cruz	#47778	1:400
**Secondary Antibodies**	**SOURCE**	**IDENTIFIER**	**DILUTION**
HRP-conjugated goat anti-rabbit	R&D Systems	HAF008	1:2000
Digital anti-Mouse HRP	KwikQuant	R1005	1:2000
Digital anti-Rabbit HRP	KwikQuant	R1006	1:2000

Original images of immunoblots

Figure 1C

TSG101

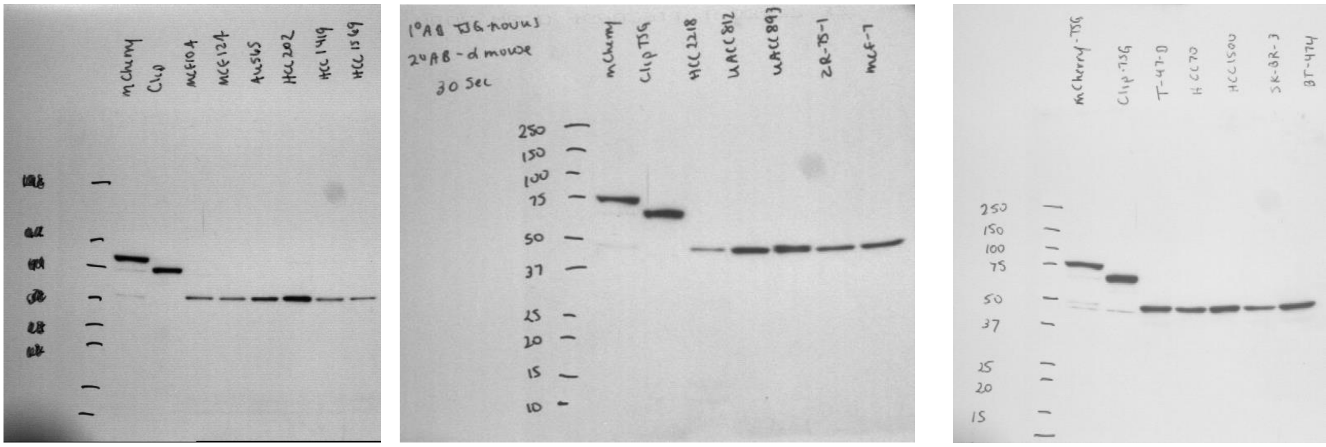

ACTB

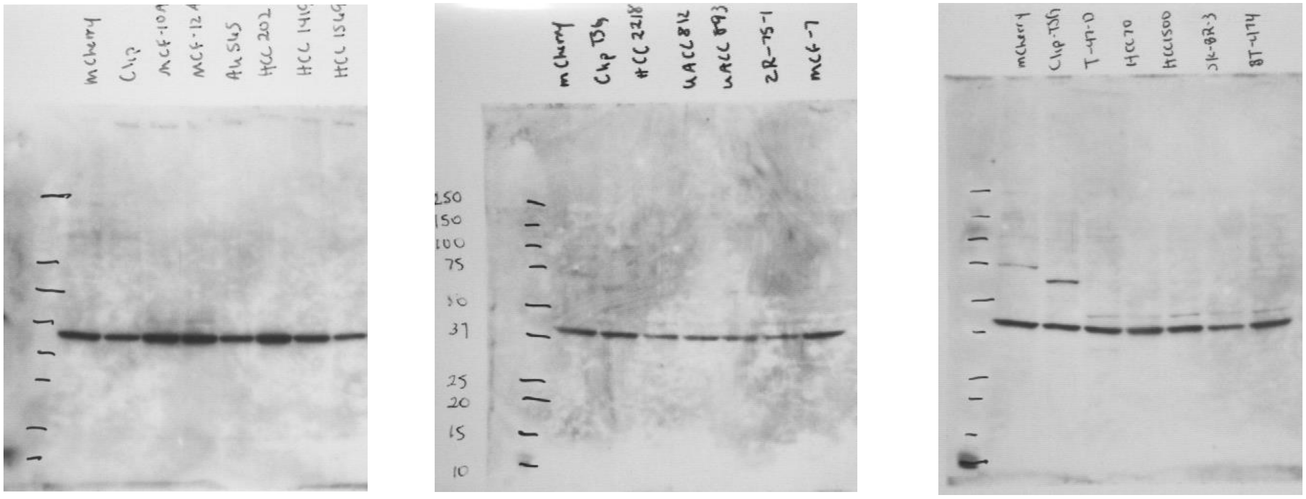

Uncropped images of immunoblots

Figure 4A

upper panel

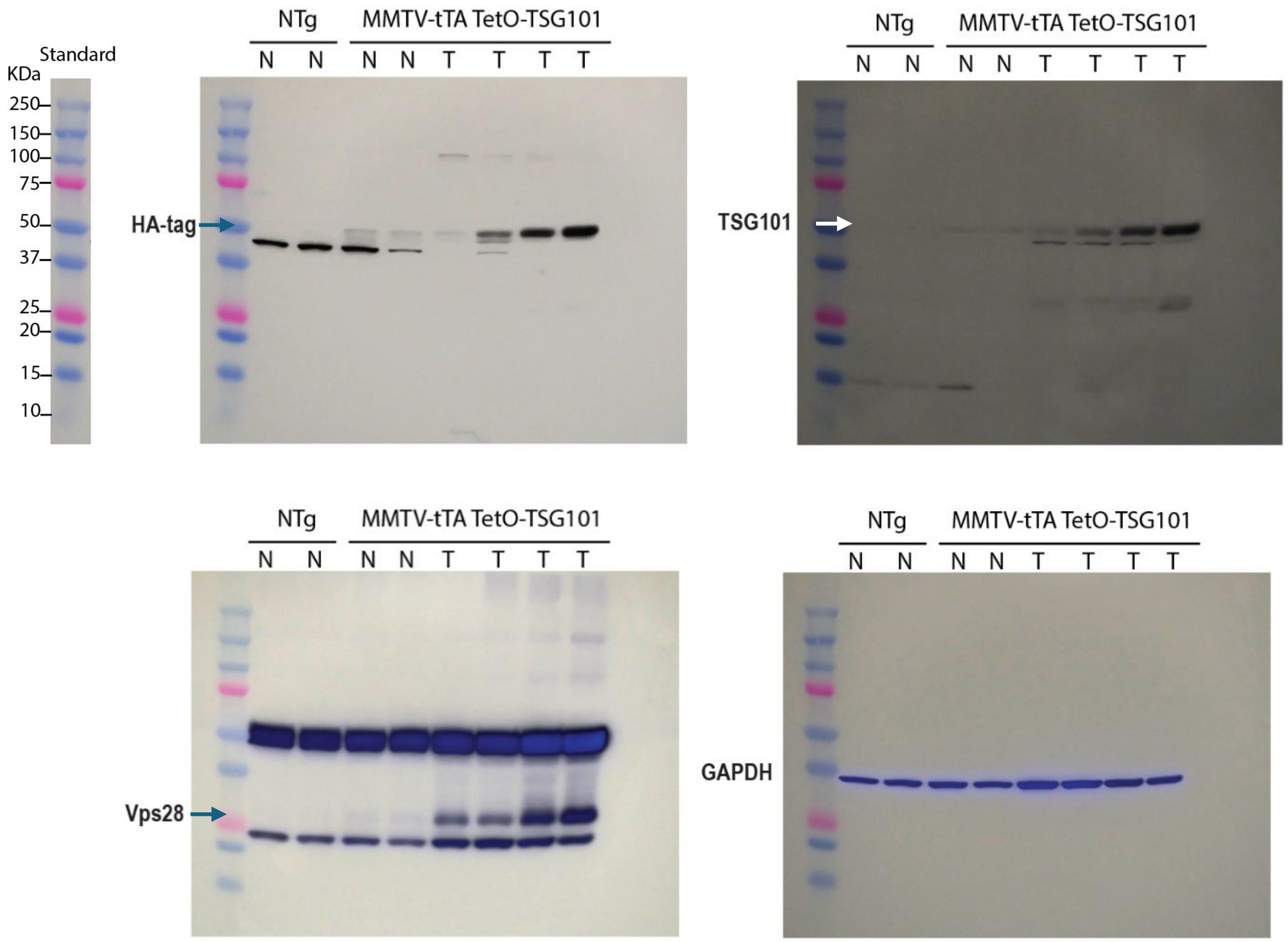

Please note that the luminescence images for each immunoblot were captured along with a brightfield image of the membrane to subsequently make the overlay with the size markers that are shown here. The overlay images may appear darker (see comparison of an example below); the captured images without the size markers were used to assemble the composite panels shown in the main figures of the manuscript.

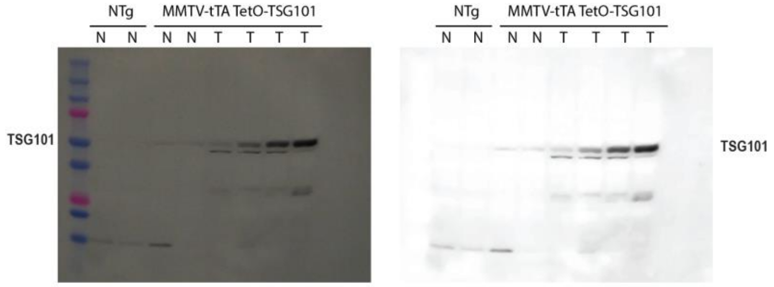

Figure 4A

lower panel

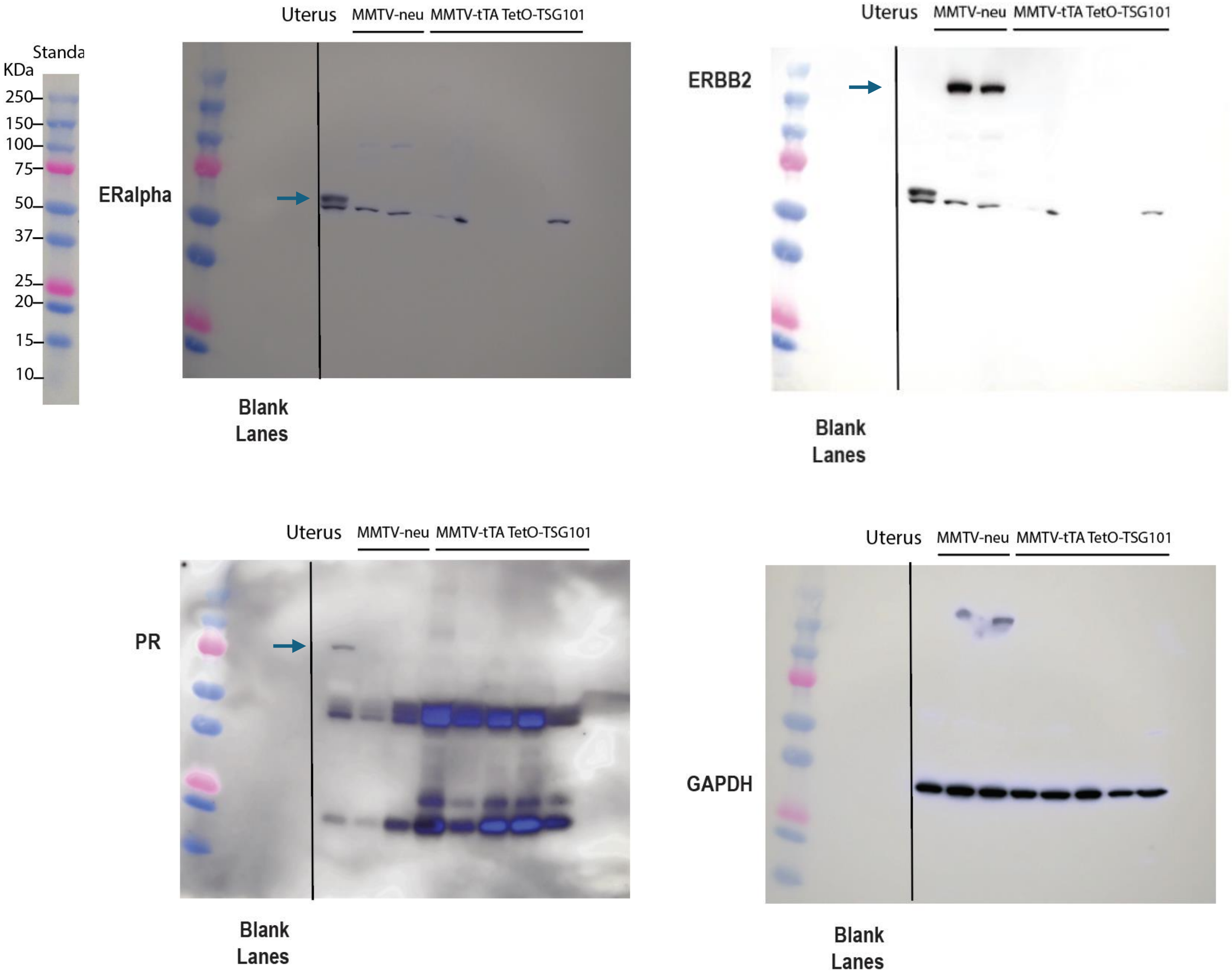

Figure 4C
left panel

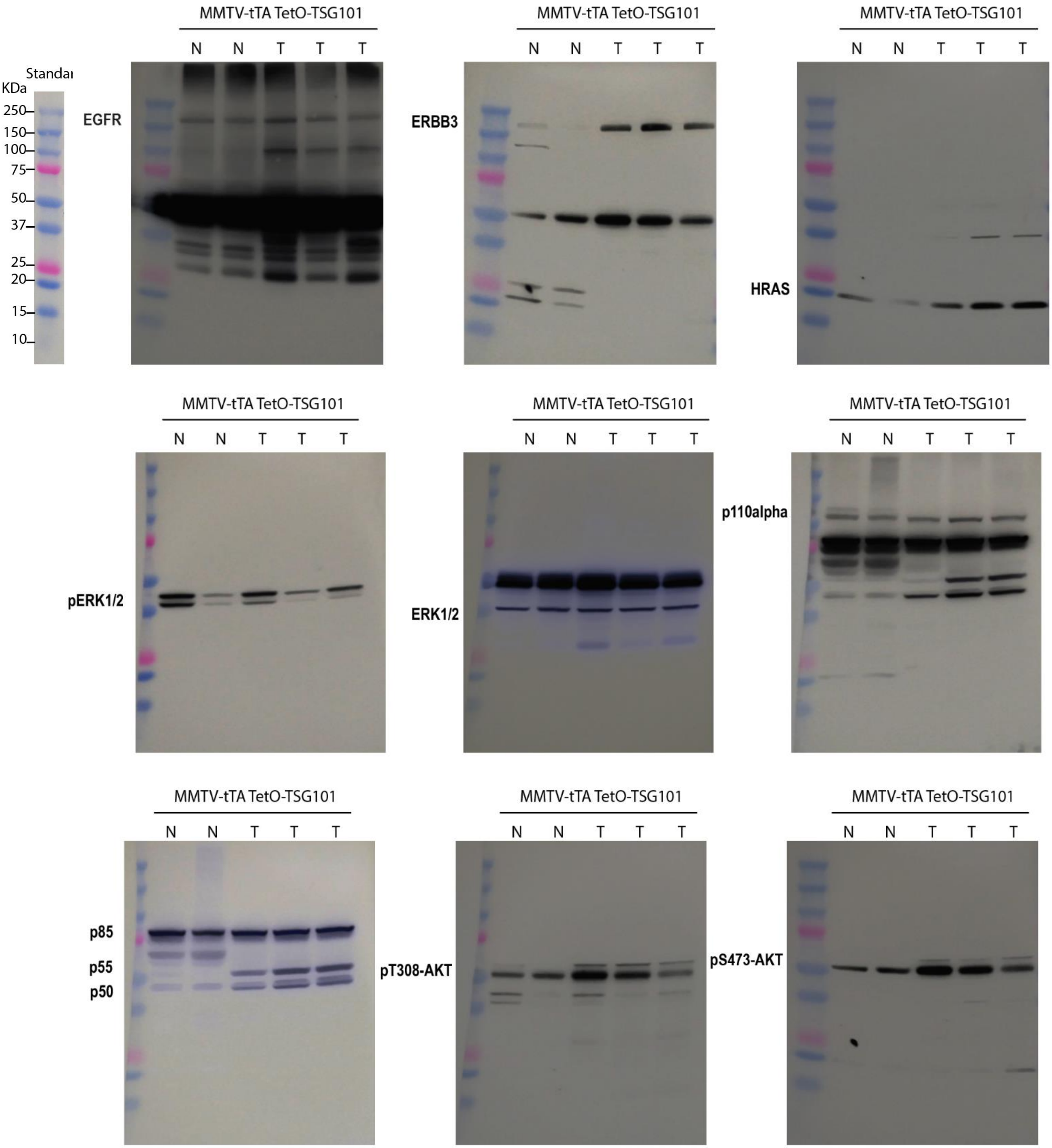

Figure 4C

left panel continued

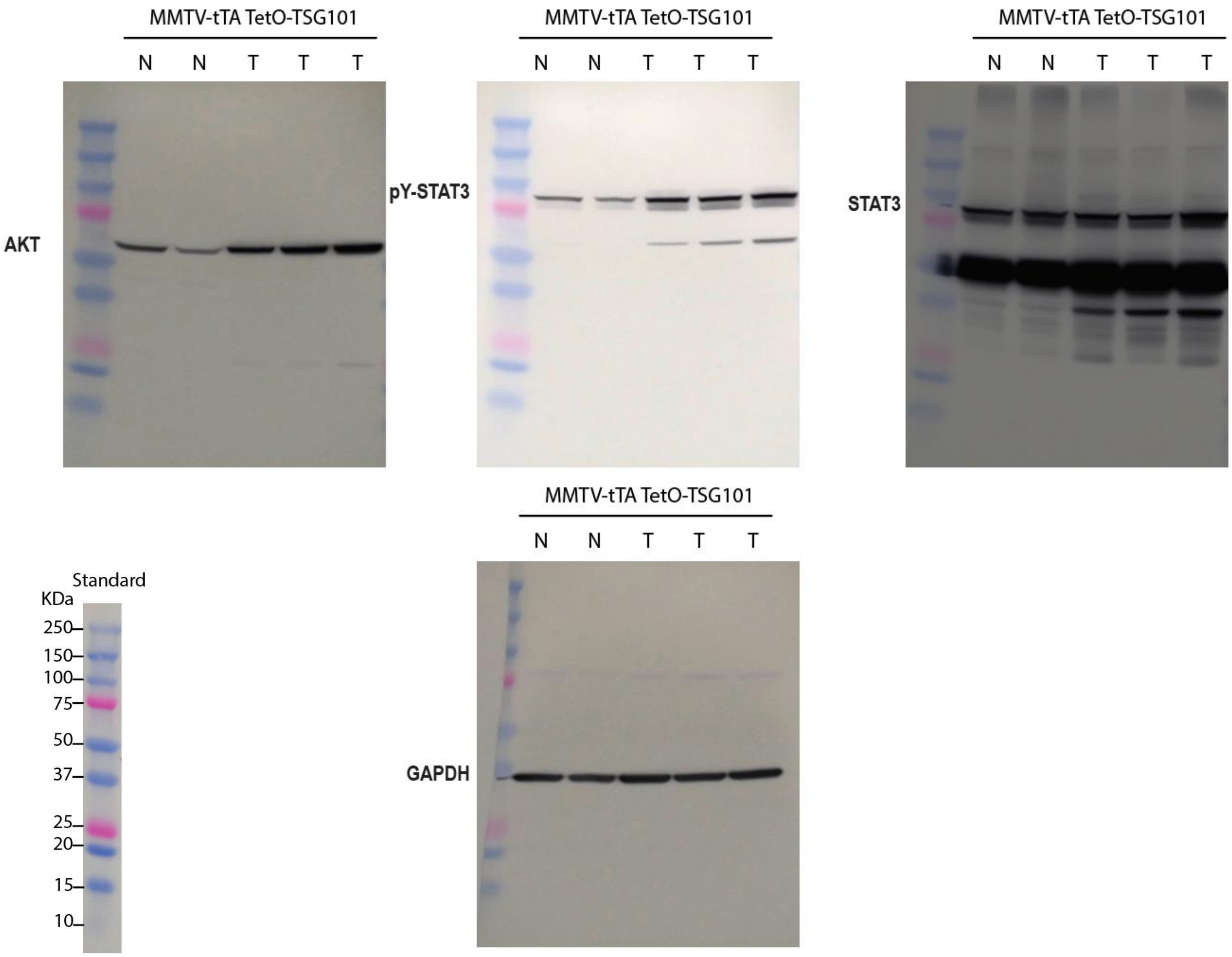

Figure 4C right upper panel

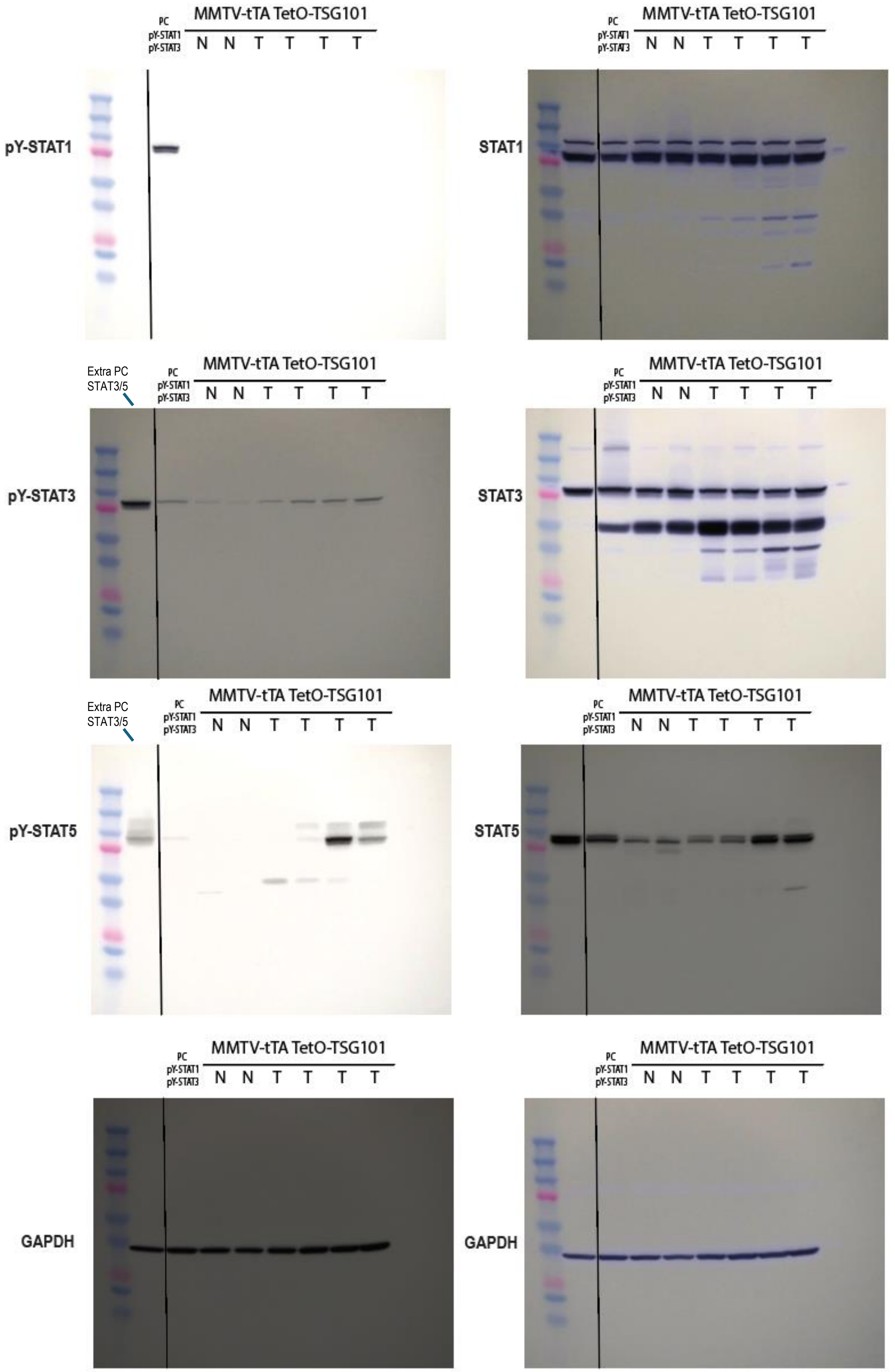

Figure 4C right lower panel

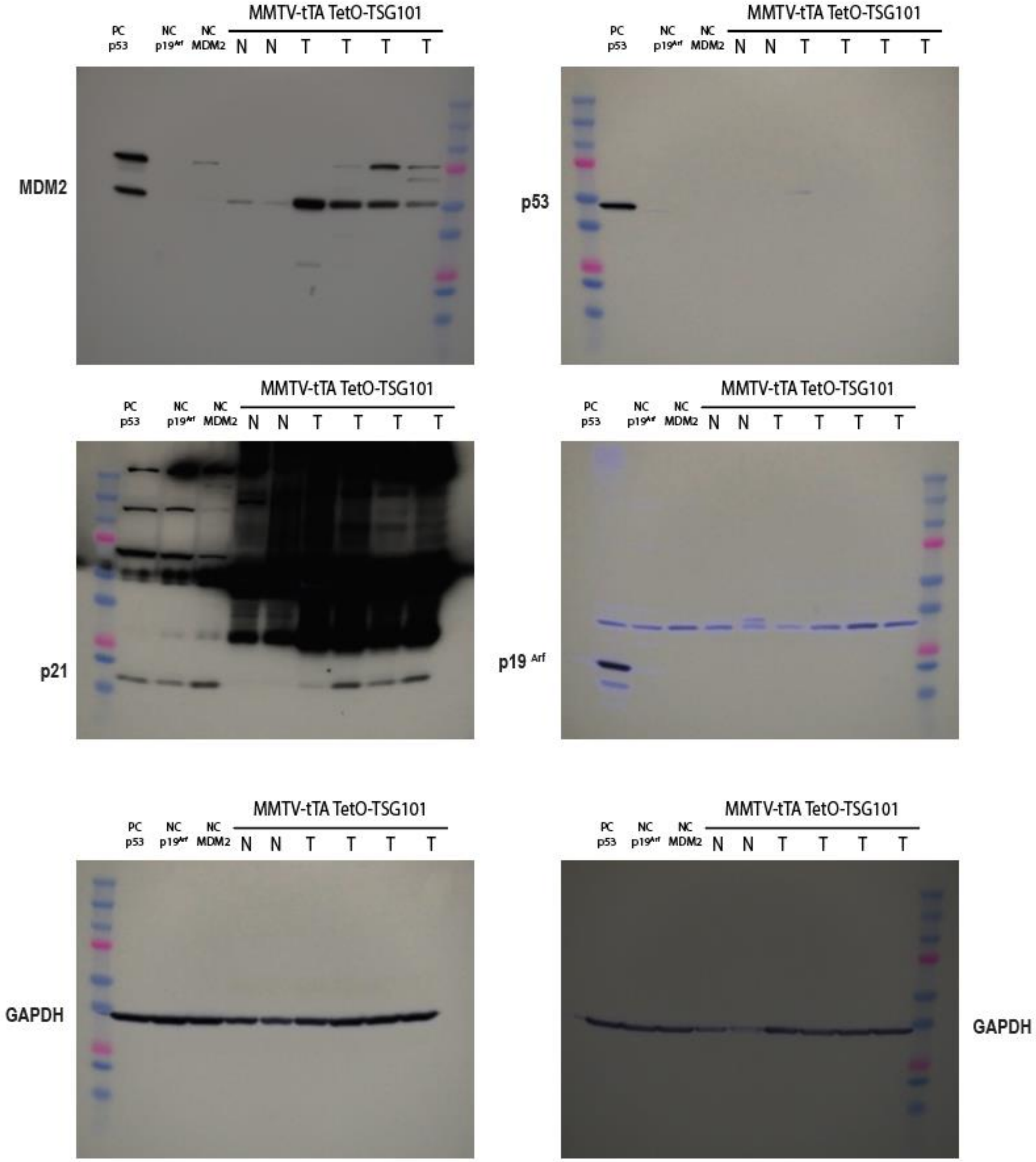

Figure 7C

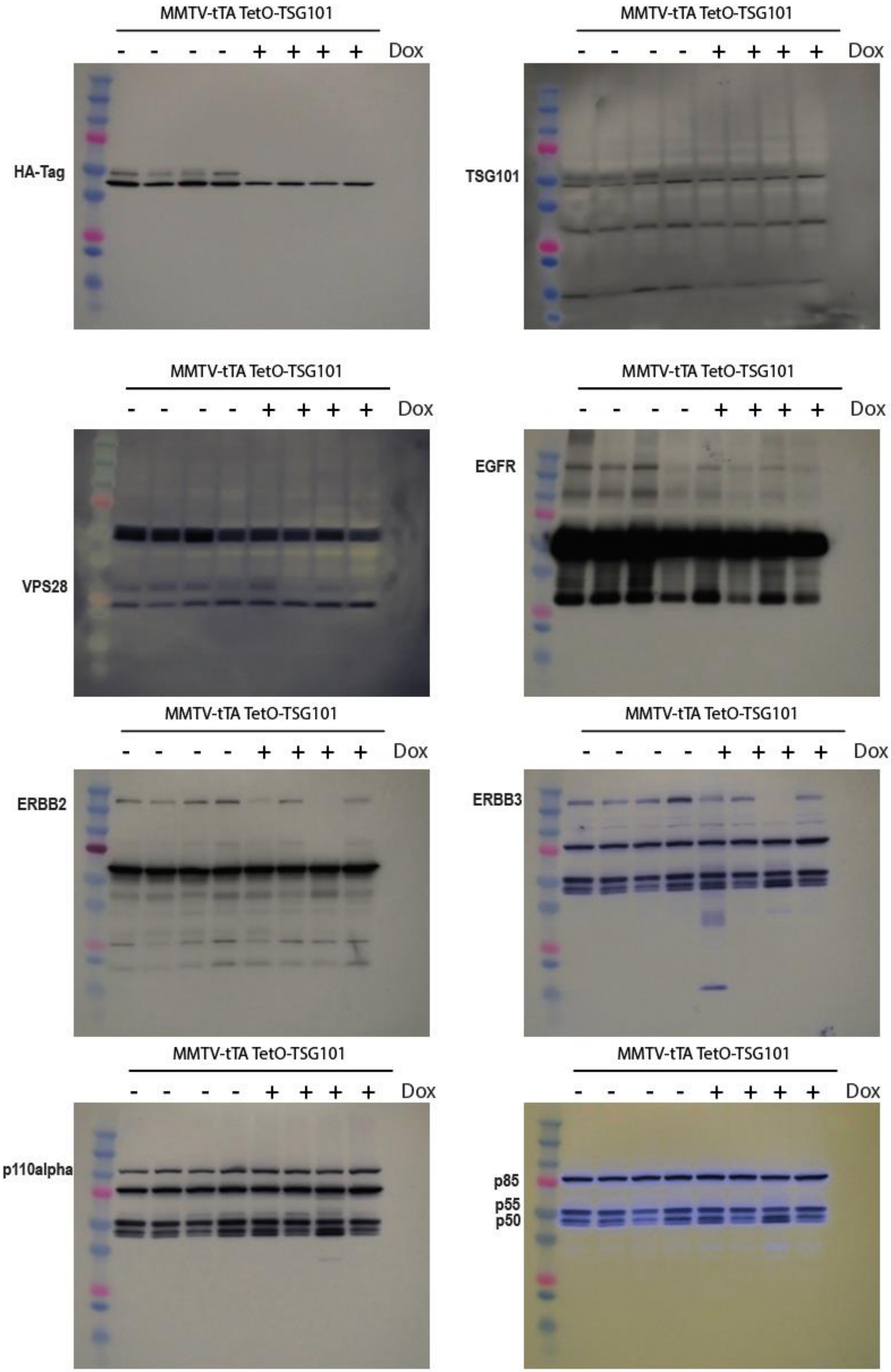
MMTV-tTA TetO-TSG101
- - - - + + + + Dox
HA-Tag
MMTV-tTA TetO-TSG101
- - - - + + + + Dox
TSG101
MMTV-tTA TetO-TSG101
- - - - + + + + Dox
VPS28
MMTV-tTA TetO-TSG101
- - - - + + + + Dox
EGFR
MMTV-tTA TetO-TSG101
- - - - + + + + Dox
ERBB2
MMTV-tTA TetO-TSG101
- - - - + + + + Dox
ERBB3
MMTV-tTA TetO-TSG101
- - - - + + + + Dox
p110alpha
MMTV-tTA TetO-TSG101
- - - - + + + + Dox
p85
p55
p50

Figure 7C continued

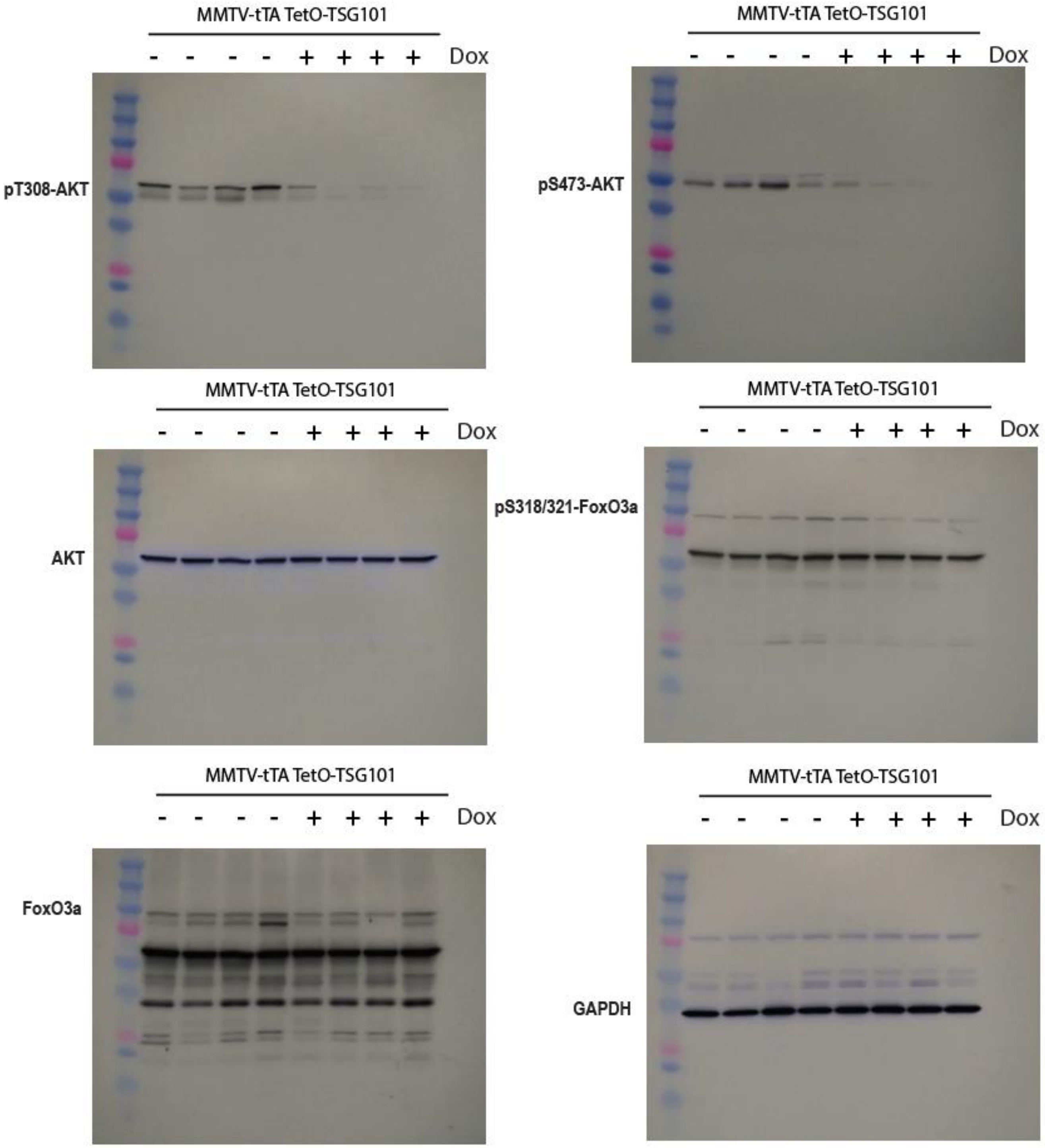

Supplement: Supplementary file 1 — Supplementary material 1. [file 13058_2025_2007_MOESM1_ESM.pdf]
